# Cytochrome *b* Divergence between Avian Sister Species Is Linked to Generation Length and Body Mass

**DOI:** 10.1371/journal.pone.0085006

**Published:** 2014-02-05

**Authors:** Caroline E. Thomson, James D. J. Gilbert, M. de L Brooke

**Affiliations:** 1 Department of Zoology, University of Cambridge, Cambridge, United Kingdom; 2 Department of Zoology, University of Oxford, Oxford, United Kingdom; 3 School of Biological Sciences, University of Sydney, New South Wales, Australia; Instituto de Higiene e Medicina Tropical, Portugal

## Abstract

It is increasingly realised that the molecular clock does not tick at a constant rate. Rather, mitochondrial mutation rates are influenced by factors such as generation length and body mass. This has implications for the use of genetic data in species delimitation. It could be that speciation, as recognised by avian taxonomists, is associated with a certain minimum genetic distance between sister taxa, in which case we would predict no difference in the cytochrome *b* divergence of sister taxa according to the species' body size or generation time. Alternatively, if what taxonomists recognise as speciation has tended to be associated with the passage of a minimum amount of time since divergence, then there might be less genetic divergence between sister taxa with slower mutation rates, namely those that are heavier and/or with longer generation times. After excluding non-flying species, we analysed a database of over 600 avian sister species pairs, and found that species pairs with longer generation lengths (which tend to be the larger species) showed less cytochrome *b* divergence. This finding cautions against using any simple unitary criterion of genetic divergence to delimit species.

## Introduction

In the face of mounting evidence that the rate of molecular evolution varies between different lineages, biologists have been increasingly obliged to abandon the simple, albeit appealing, idea of a molecular clock ticking at a constant rate [1]. As knowledge of the specifics of rate variation has grown, so the emphasis has shifted towards trying to understand the basis for variation. Several factors, which may be linked to one another, for example body size, metabolic rate, generation length and population size, have been identified as correlates of the rate of molecular evolution [2]–[4].

While body size may be correlated with the rate of molecular evolution, that correlation does not of itself shed light on the underlying cause of the correlation. First, the correlation might be mediated via generation length [5]. Species that are smaller tend to have shorter generation lengths and thus, per unit time, a higher number of DNA replication rounds within the germline and, consequently, a greater probability of replication errors [4], [6].

Second, the correlation between body size and rate of molecular evolution might be mediated via metabolic rate. Among homeotherms, such as birds, whole-body metabolic rate scales with body mass to the power of approximately ¾ and mass-specific metabolic rate to the power of - ¼ [7]–[8], but see also ref [9]. That scaling could lead to a reduced rate of mitochondrial respiration in larger animals and a reduced rate of production of mutagenic free radicals, a by-product of aerobic respiration [10]–[11]. Whether such scaling in the somatic cells also affects the germ-line cells, from where mutations will be transmitted to the next generation, remains unclear [12]–[13].

A separate hypothesis holds that smaller population sizes, which are often associated with larger body size [14], may allow more slightly deleterious mutations to drift to fixation. This leads to a prediction of an inverse relation between effective population size and rate of molecular evolution, a prediction for which Woolfit and Bromham [15] obtained support from island populations. It also leads to the possibility that larger species, whose generally smaller populations will be exposed to a greater risk of fixing deleterious mutations, may invest more in DNA copy fidelity and repair mechanisms [4], [16] - which would enhance the inverse relationship between generation time and rate of molecular evolution. This enhanced reduction of mutation rate in long-lived, as opposed to short-lived, species is the ‘longevity hypothesis’ [13], [16]. Its predictions overlap with those of the generation time hypothesis of the previous paragraph.

In the past, when the biological species concept prevailed [17], genetic distance data were not widely used to inform species delimitation. However, over the last three decades as, first, amino-acid and then nucleic acid sequence data became available, so the phylogenetic species concept has risen in prominence [18], not least because sequence data are well suited for phylogenetic reconstruction [19]. While ornithologists have sometimes struggled with how best to integrate molecular data into species diagnosis [20]–[21], such data are frequently used in practice [22]–[25]. This renders it crucial to understand factors impinging on the genetic distance between related species. If a suite of factors influences mutation rate, then this information should certainly be noted and possibly be incorporated when genetic data are used to delimit species.

In fact, it has long been recognized that there is no uniform level of genetic divergence between vertebrate species, whether allozyme data [26] and/or DNA sequence data (e.g. from cytochrome *b* [27]) are used. This is to be expected, because the times since sister taxa first diverged and then speciated will vary. That proviso acknowledged, if speciation tended to be associated with a certain minimum genetic distance between sister taxa, then we would predict no difference in the cyt *b* divergence of sister taxa according to body size or generation time. Alternatively, if speciation tended to be associated with the passage of minimum amount of time since divergence, sufficient, for example, for reproductive isolation to develop, then there might be less genetic divergence between sister taxa that are heavier and/or with longer generation times [28], because, as discussed above, these species will have experienced lower rates of molecular evolution at neutral marker sites.

Our paper addresses the possibility of a relationship between the cyt *b* divergence of avian sister taxa, the response variable in the analyses, and the average mass, mass difference and generation length of those taxa. To our knowledge, this possibility has not hitherto been investigated. We chose cyt *b* as a gene widely used in avian barcoding studies [29]. Crucially, we emphasize we are taking current species as a given, and asking whether we can identify factors that are correlated with the degree of genetic difference between those species.

## Methods

### Genetic distance values

Gene sequences were obtained from Genbank for the mitochondrial gene cyt *b*. All available sequences within a particular genus were downloaded into MEGA4 [30], provided that at least half of the species within the genus had sequences available. The consequences of using a more stringent 75% cut-off, as opposed to the 50% cut-off described, are trivial (see Results). Sequences within each genus were then aligned using ClustalW [31] within MEGA4, and were adjusted by eye. Any sequences that did not align, or were much shorter than all others, were removed. The ends of sequences were then trimmed so that all were of the same length within the alignment group.

Once the sequences were aligned, molecular phylogenies were created for the various individual genera, using both the maximum parsimony and neighbour joining methods, in order to find the sister species pairs within that genus, including the cases where a given sister species pair was already known. In many cases, published phylogenies supported the pairings. Species involved in unresolved polytomies were discarded. These genus-level phylogenies were not used in the wider phylogenetic analysis discussed below and reported in the Results.

After sister species pairs were found, the genetic distance between them was calculated, using the Tamura-Nei model [32]. The Tamura-Nei model accounts for unequal nucleotide frequencies, and different rates of nucleotide transitions and transversions. Where multiple sequences were present for either species, the average value of divergence between the species was calculated. It is worth noting that, as our comparisons involved sister species, saturation of sites is unlikely to be a serious problem. The full dataset is presented in the Table S1.

### Mass data

Data on species masses were obtained from [33]. Where a mass range rather than mean value was given for a species, the median of this range was taken. The mean value for each species was calculated if more than one value was given (i.e. separate masses provided for males and females). For analyses of average body mass, a single value was needed for each pair, rather than separate values for each species, so the mean value of the masses was calculated. Where mass data were only available for one of the species pair, then that value was taken to represent both species. However, for analyses involving differences in species' mass, we could only use those pairs where mass data were available for both species. The difference in mass was calculated and then expressed as a percentage of the mass of the heavier member of the pair.

Non-flying species, ratites and penguins, were excluded from the mass and generation time analysis since determinants of these species' masses are likely to be different to those of volant birds.

### Generation length

Species generation lengths were obtained from a database supplied by BirdLife International (http://www.birdlife.org/datazone/home) which, following [34], defines generation length as the average age of the parents of the current cohort.

### Statistical analysis

Statistical analyses were carried out in R [35] and R commander [36]. Initial histograms of the cyt *b* divergences revealed that the data were positively skewed, so the Shapiro-Wilk test for normality [37] was carried out. The data were not normally distributed, and so the square roots of the Tamura-Nei values were used for subsequent analyses.

After initial investigation using ordinary least-squares (OLS) models, we analysed the effects of mass and generation length on divergence using phylogenetic generalized least-squares models (PGLS), using the pglmEstLambda function in CAICR [38] and ape 2.3 packages [39] in R. This method gives an estimate of the strength of the phylogenetic signal within the data (the λ value: [40]) as well as estimating the effect of a given factor when the phylogeny is incorporated into the model. As with the linear analyses, the PGLS analysis used the square root of the Tamura-Nei distance for cyt *b* divergence to improve normality of residuals.

We fitted all possible combinations of our three predictor variables and their interactions, comparing models using Akaike's Information Criterion (AIC; all fitted models are shown in Table 1). Following Burnham & Anderson [41] we accepted as “top models” all those within a threshold of 2 AIC points of the minimum within the model set.

**Table 1:** AIC table and coefficients for all models fitted in PGLS analyses.

AIC Table						Coefficients													
Model	AIC	ΔAIC	RL	W_a_	λ	M	SE	D	SE	G	SE	M∶D	SE	M∶G	SE	D∶G	SE	M∶D∶G	SE
**D+G**	**−596.07**	**0.00**	**1.000**	**0.410**	**0.375**			**9.070×10^−4^**	**3.099×10^−4^**	**−0.004**	**0.001**								
M+D+G	−593.35	2.72	0.257	0.105	0.376	−2.665×10^−6^	5.197×10^−6^	8.988×10^−4^	3.107×10^−4^	−0.003	0.002								
M+D	−593.35	2.72	0.256	0.105	0.432	−8.203×10^−6^	4.210×10^−6^	8.914×10^−4^	3.108×10^−4^										
D+G+D∶G	−593.26	2.81	0.245	0.100	0.373			6.236×10^−4^	6.698×10^−4^	−0.004	0.002					3.410×10^−5^	7.145×10^−4^		
D	−592.54	3.53	0.171	0.070	0.461			9.246×10^−4^	3.112×10^−4^										
M+D+G+M∶G	−591.27	4.81	0.090	0.037	0.357	1.636e×10^−5^	1.460×10^−5^	9.426×10^−4^	3.142×10^−4^	−0.004	0.002			1.019×10^−6^	1.015×10^−6^				
G	−591.15	4.92	0.085	0.035	0.355					−0.004	0.001								
M+D+M∶D	−590.68	5.39	0.067	0.028	0.426	−1.067×10^−5^	6.017×10^−6^	7.740×10^−4^	3.727×10^−4^			1.558×10^−7^	2.720×10^−7^						
M+D+G+M∶D	−590.64	5.44	0.066	0.027	0.373	−4.997×10^−6^	6.837×10^−6^	7.909×10^−4^	3.726×10^−4^	−0.003	0.002	1.431×10^−7^	2.720×10^−7^						
M+D+G+D∶G	−590.49	5.59	0.061	0.025	0.374	−2.403×10^−6^	5.239×10^−6^	6.488×10^−4^	6.730×10^−4^	−0.004	0.002					3.017×10^−5^	7.205×10^−5^		
M	−588.78	7.29	0.026	0.011	0.417	−8.883×10^−6^	4.248×10^−6^												
M+D+G+M∶D+M∶G	−588.70	7.37	0.025	0.010	0.352	2.045×10^−5^	1.585×10^−5^	8.103×10^−4^	3.734×10^−4^	−0.004	0.002	1.804×10^−7^	2.742×10^−7^	1.104×10^−6^	1.023×10^−6^				
M+G	−588.59	7.48	0.024	0.010	0.357	3.418×0^−6^	5.242×10^−6^			−0.003	0.002								
M+D+G+M∶G+D∶G	−588.56	7.51	0.023	0.010	0.352	1.743×10^−5^	1.471×10^−5^	5.834×10^−4^	6.761×10^−4^	−0.005	0.003			1.126×10^−6^	1.030×10^−6^	4.392×10^−5^	7.317×10^−5^		
M+D+G+M∶D+D∶G	−587.64	8.43	0.015	0.006	0.372	4.402×10^−6^	7.528×10^−6^	6.824×10^−4^	6.803×10^−4^	−0.003	0.002	1.149×10^−7^	3.101×10^−7^			1.567×10^−5^	8.215×10^−5^		
1	−587.47	8.60	0.014	0.006	0.447														
M+G+M∶G	−585.88	10.19	0.006	0.003	0.342	1.157×10^−5^	1.470e×10^−5^			−0.004	0.002			6.057×10^−7^	1.018×10^−6^				
M+D+G+M∶D+M∶G+D∶G	−585.75	10.32	0.006	0.002	0.350	2.003×10^−5^	1.593×10^−5^	6.212×10^−4^	6.831×10^−4^	−0.005	0.003	1.324×10^−7^	3.108×10^−7^	1.148×10^−6^	1.032×10^−6^	2.744×10^−5^	8.294×10^−5^		
M*G*D	−583.28	12.79	0.002	0.001	0.343	1.263×10^−5^	1.940×10^−5^	9.595×10^−4^	8.490×10^−4^	−0.004	0.003	4.178×10^−7^	8.718×10^−7^	5.667×10^−7^	1.350×10^−6^	7.452×10^−6^	9.789×10^−5^	4.324×10^−8^	6.394×10^−8^

Models within 2 AIC points of the top model are given in bold. Key to symbols: M, Mean body mass for species pair; D, Mass difference of species pair, expressed as a percentage of the heavier species' mass; G, Mean generation length for species pair; RL, Model relative likelihood; W_a_, Model Akaike weight.

A phylogeny of bird species, based on genetic, behavioural and morphological data, was obtained from the Tree of Life [42]. The tree did not contain all the genera within the dataset, so the dataset was trimmed to contain only those genera that were available within the tree, and likewise the tree was trimmed to only those genera found within the dataset. Hence the dataset used in phylogenetic analyses was a subset of the original. Branch lengths on the phylogeny were unknown and were therefore arbitrarily set to 1.

## Results

The mean Tamura-Nei divergence of cyt *b* between sister species pairs was 0.0491±s.d. 0.0334 (5th–95th percentiles = 0.005–0.109: *n* = 633 species pairs; see Table S1). If the analysis is restricted to the smaller sample of those genera where sequence data were available for at least 75% of species within the genus (see Methods), then the mean Tamura-Nei distance barely changes (mean = 0.0492±0.0336: *n* = 512). For this reason, all results presented henceforth are based on the less stringent 50% cut-off outlined in the Methods.

**Figure 1:**
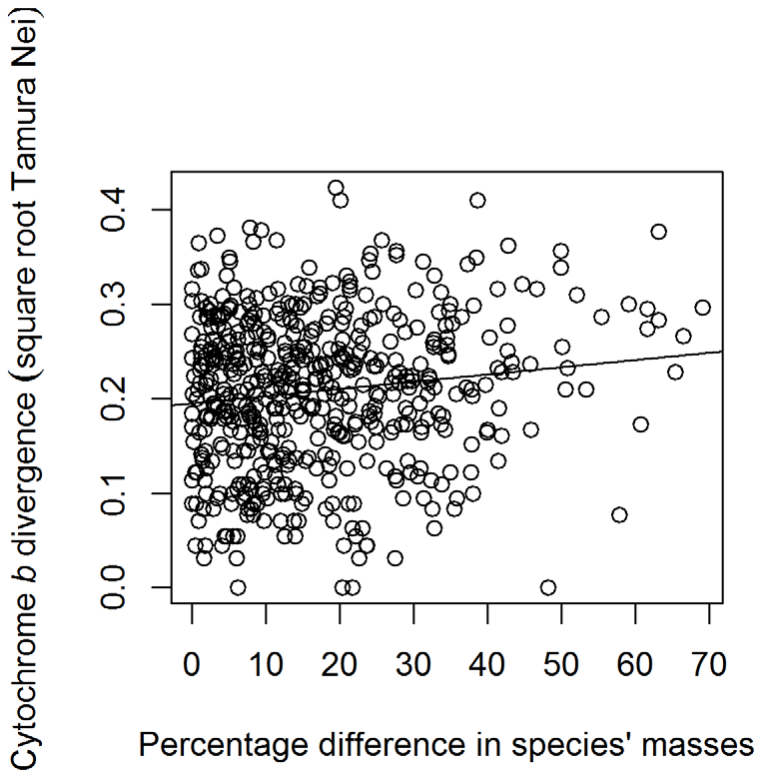
Scatterplot of the effect of the percentage difference in species' mass in each pair ( = (absolute difference in mass/mass of the heavier species)*100) on the cyt *b* divergence. There is a significant positive relationship (linear model – y = 0.1948+0.00075x, adjusted R^2^ = 0.015, *F*_1,548_ = 9.29, *p* = 0.002).

**Figure 2:**
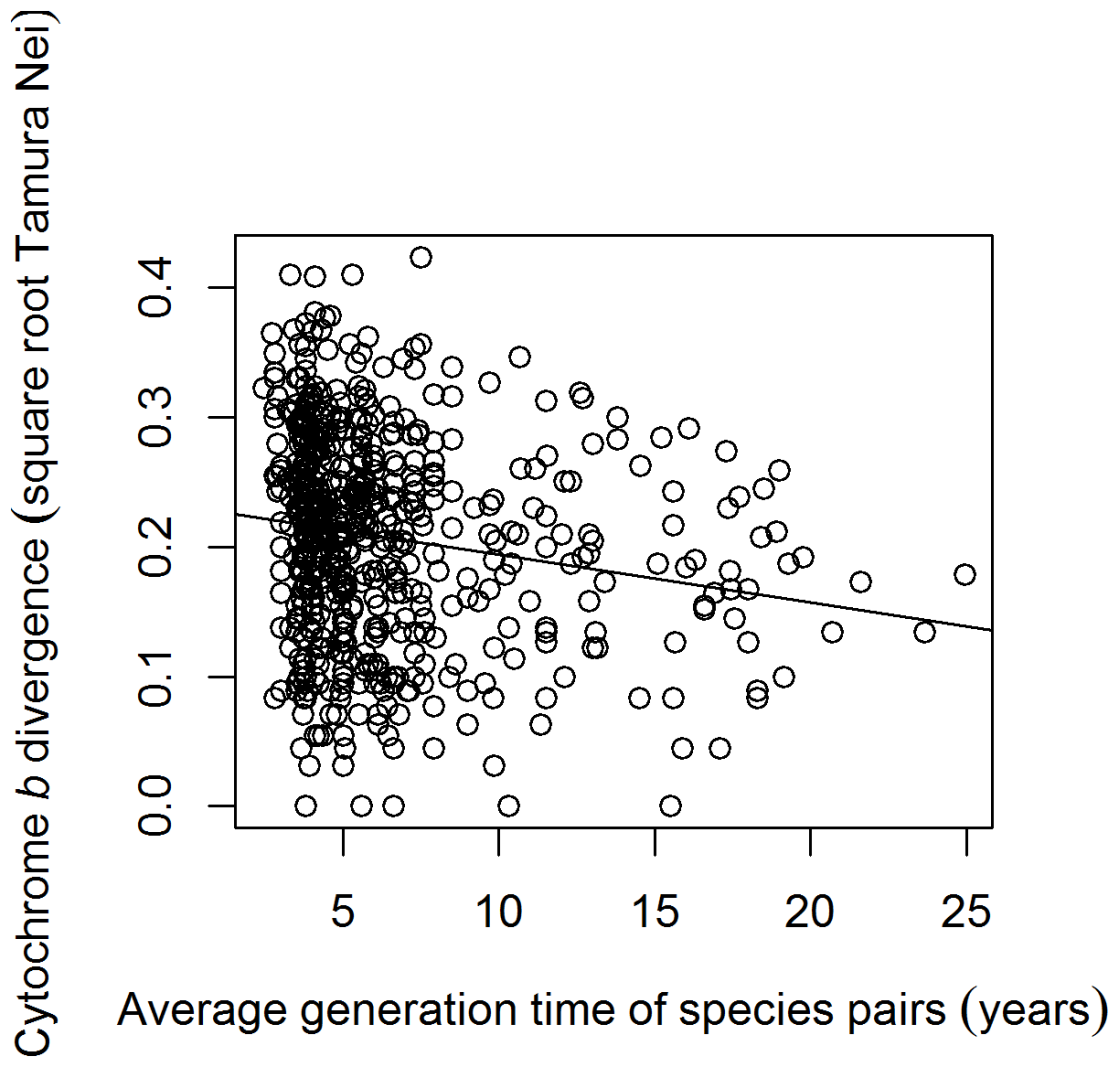
Scatterplot of the effect of generation time of species pairs on the cyt *b* divergence. There is a significant negative relationship (linear model – y = 0.2239–0.0026x, adjusted R^2^ = 0.017, *F*_1,622_ = 11.67, *p*<0.001).

All models and their associated coefficients are given in Table 1. There was a single clear top model, which included only two predictor variables: percentage mass difference and generation length (ΔAIC to the next best model = 2.72). Phylogenetic signal was strong in this model (λ = 0.375, test of λ versus 0, χ^2^ = 19.286, *p* = 1.125×10^−5^). According to this model, an increase of 10 percent in the mass difference between the two members of a species pair was associated with an increase of approximately 0.009 in their square-root transformed cyt *b* divergence (Fig. 1; dropping “mass difference” from top model, PGLS, *F*_1,274_ = 8.892, *p* = 0.003). In contrast, an increase of one year in the average generation length of a species pair was associated with a decrease of 0.004 in the square-root transformed cyt *b* divergence between the two species (Fig. 2; dropping “generation length” from top model, PGLS, *F*_1,274_ = 6.997, *p* = 0.009).

**Figure 3:**
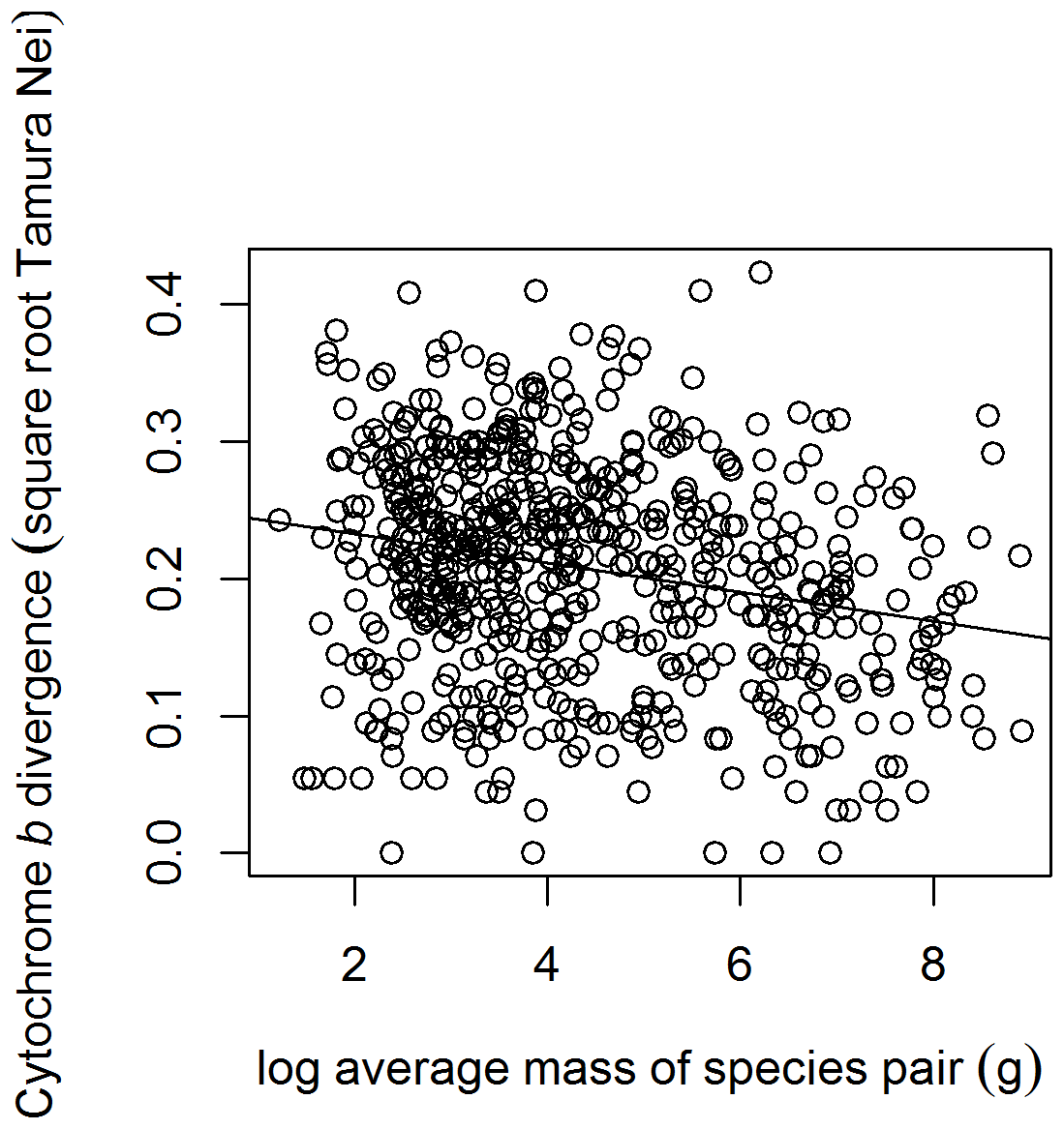
Scatterplot of the effect of the average mass of a species pair on the cyt *b* divergence, excluding all flightless species. There is a significant negative correlation between the variables (linear model – y = 0.2118 - 0.000011x, adjusted R^2^ = 0.017, *F*_1,622_ = 12.00, *p*<0.001).

Superficially, there was also what appeared to be a negative relationship between the average mass of a volant species pair and the cyt *b* divergence of the two species, even though this variable did not appear in the top model. Although this relationship was significant in a single-factor analysis under OLS regression (Fig. 3), incorporating phylogenetic information greatly reduced its significance (PGLS; *F*_1,275_ = 4.339, *p* = 0.038, λ = 0.417, test of λ versus 0, χ^2^ = 14.749, *p* = 0.0001), consistent with a general pattern of strong phylogenetic conservatism in body mass [43]. When the other important predictor variables were incorporated, the explanatory power of body mass became non-significant (dropping “body mass” from the “M+D+G” model, PGLS, *F*_1,273_ = 3.257, *p* = 0.072).

Taken together, we interpret these results as supporting the idea that generation length and percentage mass difference have independent effects on cyt *b* difference, but mass *per se* does not, at least when phylogeny is accounted for.

## Discussion

Our principal finding was that the cyt *b* divergence between avian sister taxa decreased as generation length increased. Divergence also decreased as body mass increased, but there is no strong and conclusive evidence that this latter effect was either independent of generation length, to which mass is collinearly related [28], or robust to phylogenetic correction. Thus while speciation is associated with the accumulation of some minimal amount of genetic divergence in neutral markers such as cyt *b*, other factors bear on the genetic divergence between recognised species and must be borne in mind when genetic data are used for species diagnosis [22]–[25]. Therefore, our findings caution against the simple expectation that there might be a uniform genetic divergence between sister species, particularly when comparison is made between species pairs drawn from different lineages.

Cyt *b* divergence was positively associated with the percentage difference in body size of species (Fig. 1). This association was to be expected, as species that have diverged proportionately more in body size are likely to have speciated in the more distant past, accumulating a greater number of changes in cyt *b* sequence. That would remain true whether the molecular clock ‘ticks’ at a rate proportional to absolute time, generation time or metabolic rate.

While percentage difference in mass between sister taxa is related to their cyt *b* divergence, mass *per se* has limited effect. This suggests that mass-specific metabolic rate, allometrically related to mass, has limited effect on substitution rates and divergence [10], [11], a negative result supporting those previously obtained [6], [12]. However, our conclusion must be cautious as we did not analyse actual metabolic rates.

There was a decrease in the divergence between species as their generation length increased (Fig. 2). We therefore tentatively suggest that speciation is typically recognised by taxonomists after the passage of a certain amount of time representing, on average, fewer generations in larger species, and more generations in smaller species. Those species with shorter generation lengths may have about the same proportion of DNA changes per generation as those with longer generations, and therefore more changes will accumulate per year in the species with shorter generation times [10]. However the amount of variance explained was very low, about two percent (Fig. 2, legend). Superior DNA repair in the longer-lived species (see Introduction) could contribute to the low amount of variance explained, as could the fact that the gene used in this study, cyt *b*, was mitochondrial. Such mitochondrial genes may show a weaker correlation with generation time than nuclear DNA, as there is a larger and more variable number of duplications of mitochondrial genomes per generation than of nuclear genomes [44]–[45].

Lanfear et al. [46] reported that rates of molecular evolution of protein-coding nuclear genes were positively correlated with rates of diversification in various avian lineages. If mutation promotes speciation, then it is tempting to conclude that the difference between the cyt *b* of sister species might be *lower* in fast-mutating lineages of smaller taxa. However that need not be the case. If a certain minimum amount of time is required to elapse before diverging lineages are recognised as species by taxonomists, then the smaller species pairs with shorter generation times might show *higher* divergence, as we find.

Implicit in our study is an assumption that the scientific behaviour of avian taxonomists is similar across the range of bird weights. Our results could be explained if cryptic species, awaiting ‘splitting’, were disproportionately represented among smaller species of low body mass and short generation time. Objectively excluding this possibility would be extremely taxing and we merely observe that avian taxonomy at the species level remains in a state of flux from the smallest *Phylloscopus* warblers (<10 g: [47]) to large albatrosses (c. 5 kg; [22], [48]).

The fact that a signal indicating effects of body mass and generation length on the extent of genetic divergence between sister taxa can be recovered at all is perhaps remarkable. It confirms speciation as an ongoing biological process that continues to create problems for species delimitation.

## Supporting Information

Table S1 The Table shows the 633 sister species pairs used in the analysis, and the cytochrome *b* divergence between the pairs.
(DOC)

## References

[1] Brown WM, George M, Wilson AC (1979) Rapid evolution of animal mitochondrial DNA. P Natl Acad Sci USA 76: 1967–1971. 10.1073/pnas.76.4.1967 PMC383514 109836

[2] Mooers AO, Harvey PH (1994) Metabolic rate, generation time, and the rate of molecular evolution in birds. Mol Phylogenet Evol 3: 344–350. 7697191 10.1006/mpev.1994.1040

[3] Nunn GB, Stanley SE (1998) Body size effects and rates of cytochrome b evolution in tube-nosed seabirds. Mol Biol Evol 15: 1360–1371. 9787440 10.1093/oxfordjournals.molbev.a025864

[4] Bromham L (2009) Why do species vary in their rate of molecular evolution? Biol Lett 5: 401–404. 19364710 10.1098/rsbl.2009.0136 PMC2679939

[5] Laird CD, McConaughty BL, McCarthy BJ (1969) Rate of fixation of nucleotide substitutions in evolution. Nature 224: 149–154. 5343515 10.1038/224149a0

[6] Bromham L, Rambaut A, Harvey PH (1996) Determinants of rate variation in mammalian DNA sequence evolution. J Mol Evol 43: 610–621. 8995058 10.1007/BF02202109

[7] Gillooly JF, Brown JH, West GB, Savage VM, Charnov EL (2001) Effects of size and temperature on metabolic rate. Science 293: 2248–2251. 11567137 10.1126/science.1061967

[8] Gillooly JF, Allen AP, West GB, Brown JH (2005) The rate of DNA evolution: Effects of body size and temperature on the molecular clock. P Natl Acad Sci USA 102: 140–145. 10.1073/pnas.0407735101 PMC544068 15618408

[9] Kolokotrones T, Savage V, Deeds EJ, Fontana W (2010) Curvature in metabolic scaling. Nature 464: 753–756. 20360740 10.1038/nature08920

[10] Martin AP, Palumbi SR (1993) Body size, metabolic rate, generation time, and the molecular clock. P Natl Acad Sci USA 90: 4087–4091. 10.1073/pnas.90.9.4087 PMC46451 8483925

[11] Wallace DC (2005) A mitochondrial paradigm of metabolic and degenerative diseases, aging, and cancer: A dawn for evolutionary medicine. Annu Rev Genet 39: 359–407. 16285865 10.1146/annurev.genet.39.110304.095751 PMC2821041

[12] Lanfear R, Thomas JA, Welch JJ, Brey T, Bromham L (2007) Metabolic rate does not calibrate the molecular clock. P Natl Acad Sci USA 104: 15388–15393. 10.1073/pnas.0703359104 PMC2000532 17881572

[13] Galtier N, Jobson RW, Nabholz B, Glémin S, Blier PU (2009) Mitochondrial whims: metabolic rate, longevity and the rate of molecular evolution. Biol Lett 5: 413–416. 19324654 10.1098/rsbl.2008.0662 PMC2679905

[14] Blackburn TM, Gaston KJ (1999) The relationship between animal abundance and body size: A review of the mechanisms. Adv Ecol Res 28: 181–210.

[15] Woolfit M, Bromham L (2005) Population size and molecular evolution on islands. P Roy Soc B 272: 2277–2282. 10.1098/rspb.2005.3217 PMC1560185 16191640

[16] Nabholz B, Glémin S, Galtier N (2008) Strong variations of mitochondrial mutation rate across mammals - the longevity hypothesis. Mol Biol Evol 25: 120–130. 17998254 10.1093/molbev/msm248

[17] Mayr E (1942) Systematics and the Origin of Species, from the Viewpoint of a Zoologist. Cambridge: Harvard University Press.

[18] Cracraft J (1992) The species of the birds-of-paradise (Paradiseidae); applying the phylogenetic species concept to a complex pattern of diversification. Cladistics 8: 1–43. 10.1111/j.1096-0031.1992.tb00049.x 34929952

[19] Avise JC (2004) Molecular markers, natural history, and evolution, 2^nd^ edition. Sunderland, Massachussetts: Sinauer.

[20] Helbig AJ, Knox AG, Parkin DT, Sangster G, Collinson M (2002) Guidelines for assigning species rank. Ibis 144: 518–525.

[21] Tobias JA, Seddon N, Spottiswoode CN, Pilgrim JD, Fishpool LDC, et al (2010) Quantitative criteria for species delimitation. Ibis 152: 724–746.

[22] Abbott CL, Double MC (2003) Phylogeography of shy and white-capped albatrosses inferred from mitochondrial DNA sequences: implications for population history and taxonomy. Mol Ecol 12: 2747–2758. 12969477 10.1046/j.1365-294x.2003.01944.x

[23] Kerr KCR, Stoeckle MY, Dove CJ, Weight LA, Francis CM, et al (2007) Comprehensive DNA barcode coverage of North American birds. Mol Ecol Notes 7: 535–543. 18784793 10.1111/j.1471-8286.2007.01670.x PMC2259444

[24] Efe MA, Tavares ES, Baker AJ, Bonatto SL (2009) Multigene phylogeny and DNA barcoding indicate that the Sandwich tern complex (*Thalasseus sandvicensis*, Laridae, Sternini) comprises two species. Mol Phylogenet Evol 52: 263–267. 19348954 10.1016/j.ympev.2009.03.030

[25] Kerr KCR, Dove CJ (2013) Delimiting shades of gray: phylogeography of the Northern Fulmar, *Fulmarus glacialis* . Ecol Evol 3: 1915–1930. 23919139 10.1002/ece3.597 PMC3728934

[26] Avise AC, Aquadro CF (1982) A comparative summary of genetic distances in the vertebrates. Evol Biol 15: 151–184. 10.1093/oxfordjournals.molbev.a025875 12572611

[27] Johns GC, Avise JC (1998) A comparative summary of genetic distances in the vertebrates from the mitochondrial cytochrome *b* gene. Mol Biol Evol 15: 1481–1490. 12572611 10.1093/oxfordjournals.molbev.a025875

[28] Bennett PM, Owens IPF (2002) Evolutionary ecology of birds: life histories, mating systems, and extinction. Oxford, UK: Oxford University Press.

[29] Aliabadian M, Kaboli M, Nijman V, Vences M (2009) Molecular identification of birds: performance of distance-based DNA barcoding in three genes to delimit parapatric species. PLoS ONE 4: e4119. 19127298 10.1371/journal.pone.0004119 PMC2612741

[30] Tamura K, Dudley J, Nei M, Kumar S (2007) MEGA4: Molecular Evolutionary Genetics Analysis (MEGA) software version 4.0. Mol Biol Evol 24: 1596–1599. 17488738 10.1093/molbev/msm092

[31] Thompson JD, Higgins DG, Gibson TJ (1994) CLUSTAL W: improving the sensitivity of progressive multiple sequence alignments through sequence weighting, position specific gap penalties and weight matrix choice. Nucleic Acids Res 22: 4673–4680. 7984417 10.1093/nar/22.22.4673 PMC308517

[32] Tamura K, Nei M (1993) Estimation of the number of nucleotide substitutions in the control region of mitochondrial DNA in humans and chimpanzees. Mol Biol Evol 10: 512–526. 8336541 10.1093/oxfordjournals.molbev.a040023

[33] Dunning JB (2008) CRC handbook of avian body masses, 2^nd^ Ed. Boca Raton FL: CRC Press.

[34] IUCN (World Conservation Union) (2001) IUCN Red List Categories and Criteria: version 3.1. Gland, Switzerland & Cambridge, United Kingdom: IUCN SSC.

[35] R Development Core Team (2010) R: A Language and Environment for Statistical Computing. Vienna: R Foundation for Statistical Computing.

[36] Fox J, Andronic L, Ash M, Bouchet-Valet M, Boye T, et al. (2011) R commander package for R, version 1.8-1. URL: cran.r-project.org/web/packages/Rcmdr; 2011.

[37] Shapiro SS, Wilk MB (1965) An analysis of variance test for normality (complete samples). Biometrika 52: 591–611.

[38] Orme D, Freckleton R, Thomas G, Petzoldt T, Fritz S (2009) CAIC: Comparative Analyses using Independent Contrasts. R package version 1.0.4-94/r94. http://R-Forge.R-project.org/projects/caic/

[39] Paradis E, Claude J, Strimmer K (2004) APE: analyses of phylogenetics and evolution in R language. Bioinformatics 20: 289–290. 14734327 10.1093/bioinformatics/btg412

[40] Freckleton RP, Harvey PH, Pagel MD (2002) Phylogenetic analysis and comparative data: a test and review of evidence. Am Nat 160: 712–726. 18707460 10.1086/343873

[41] Burnham KP, Anderson DR (2002) Model selection and multimodel inference: a practical information-theoretic approach. 2nd Edition. New York, USA: Springer-Verlag,

[42] Mindell DP, Brown JW (2005) Neornithes. Modern Birds. Version 14 December 2005 (under construction). http://tolweb.org/Neornithes/15834/2005.12.14 in The Tree of Life Web Project, http://tolweb.org/

[43] Blomberg SP, Garland T, Ives AR (2003) Testing for phylogenetic signal in comparative data: behavioral traits are more labile. Evolution 57: 717–745. 12778543 10.1111/j.0014-3820.2003.tb00285.x

[44] Graur D, Li W-H (2000) Fundamentals of Molecular Evolution. Sunderland, MA: Sinauer.

[45] Lynch M, Koskella B, Schaack S (2006) Mutation pressure and the evolution of organelle genomic architecture. Science 311: 1727–1730. 16556832 10.1126/science.1118884

[46] Lanfear R, Ho SHW, Love D, Bromham L (2010) Mutation rate is linked to diversification in birds. P Natl Acad Sci USA 107: 20423–20428. 10.1073/pnas.1007888107 PMC2996675 21059910

[47] Helbig AJ, Martens J, Seibold I, Henning F, Schottler B, et al (1996) Phylogeny and species limits in the Palaearctic chiffchaff Phylloscopus collybita complex: Mitochondrial genetic differentiation and bioacoustic evidence. Ibis 138: 650–666.

[48] Brooke M (2004) Albatrosses and Petrels across the World. Oxford, UK: Oxford University Press.

