# Supplementary material for: Cytochrome b Divergence between Avian Sister Species Is Linked to Generation Length and Body Mass
Source: PLoS One. 2014 Feb 5;9(2):e85006. doi: 10.1371/journal.pone.0085006 (PMC3914784; doi:10.1371/journal.pone.0085006)
Supplement: Table S1 — The Table shows the 633 sister species pairs used in the analysis, and the cytochrome b divergence between the pairs. (DOC) [file pone.0085006.s001.doc]

Supplementary Table Thomson et al.

The Table shows the 633 sister species pairs used in the analysis, and the Cytochrome *b* divergence between the pairs. Ratites and penguins were excluded from the mass and generation time analyses. Where no value is entered in a % mass difference cell, then a mass was available for only one species of the pair, and the percent difference could not be calculated (see main text).

|  | **Order** | **Family** | ***Genus*** | ***Species*** | **Cytb Tamura Nei** | **Average mass of pair (g)** | **% mass difference** | **Generation time (y)** |
| --- | --- | --- | --- | --- | --- | --- | --- | --- |
| 1 | Passeriformes | Icteridae | *Agelaius* | *phoeniceus* | 0.024 | 50.25 | 8.21 | 6.2 |
|  | Passeriformes | Icteridae | *Agelaius* | *assimilis* |  |  |  | 6 |
| 2 | Passeriformes | Parulidae | *Basileuterus* | *luteoviridis* | 0.061 | 12.4 | 12.74 | 3.9 |
|  | Passeriformes | Parulidae | *Basileuterus* | *signatus* |  |  |  | 3.9 |
| 3 | Passeriformes | Sylviidae | *Bradypterus* | *cinnamomeus* | 0.087 | 18.57 | 11.81 | 3.5 |
|  | Passeriformes | Sylviidae | *Bradypterus* | *lopezi* |  |  |  | 3.5 |
| 4 | Passeriformes | Thamnophilidae | *Cercomacra* | *nigrescens* | 0.083 | 16.75 | 2.94 | 6.6 |
|  | Passeriformes | Thamnophilidae | *Cercomacra* | *serva* |  |  |  | 6.6 |
| 5 | Passeriformes | Certhiidae | *Certhia* | *brachydactyla* | 0.092 | 8.15 | 1.22 | 3.3 |
|  | Passeriformes | Certhiidae | *Certhia* | *americana* |  |  |  | 3.3 |
| 6 | Passeriformes | Muscicapidae | *Cossypha* | *albicapilla* | 0.117 | 47.6 | 37.26 | 5.4 |
|  | Passeriformes | Muscicapidae | *Cossypha* | *niveicapilla* |  |  |  | 5.4 |
| 7 | Passeriformes | Tyrannidae | *Empidonax* | *alnorum* | 0.042 | 13.17 | 5.22 | 3.8 |
|  | Passeriformes | Tyrannidae | *Empidonax* | *traillii* |  |  |  | 3.9 |
| 8 | Passeriformes | Tyrannidae | *Empidonax* | *affinis* | 0.007 | 10.85 | 7.96 | 3.7 |
|  | Passeriformes | Tyrannidae | *Empidonax* | *oberholseri* |  |  |  | 3.8 |
| 9 | Passeriformes | Thamnophilidae | *Epinecrophylla* | *haematonota* | 0.095 | 9.05 | 7.45 | 4.5 |
|  | Passeriformes | Thamnophilidae | *Epinecrophylla* | *leucophthalma* |  |  |  | 4.5 |
| 10 | Passeriformes | Muscicapidae | *Ficedula* | *albicollis* | 0.042 | 13.3 | 8.63 | 3.9 |
|  | Passeriformes | Muscicapidae | *Ficedula* | *hypoleuca* |  |  |  | 4.2 |
| 11 | Passeriformes | Alaudidae | *Galerida* | *cristata* | 0.073 | 40 | 8.61 | 3.8 |
|  | Passeriformes | Alaudidae | *Galerida* | *theklae* |  |  |  | 3.8 |
| 12 | Passeriformes | Sylviidae | *Hippolais* | *icterina* | 0.075 | 12.1 | 16.67 | 4 |
|  | Passeriformes | Sylviidae | *Hippolais* | *polyglotta* |  |  |  | 4 |
| 13 | Passeriformes | Timaliidae | *Illadopsis* | *puveli* | 0.096 | 42.7 | 23.55 | 5.8 |
|  | Passeriformes | Timaliidae | *Illadopsis* | *rufescens* |  |  |  | 5.8 |
| 14 | Passeriformes | Muscicapidae | *Luscinia* | *luscinia* | 0.045 | 21.55 | 18.91 | 4.2 |
|  | Passeriformes | Muscicapidae | *Luscinia* | *megarhynchos* |  |  |  | 4.2 |
| 15 | Passeriformes | Meliphagidae | *Melithreptus* | *albogularis* | 0.099 | 12.55 | 21.35 | 5.7 |
|  | Passeriformes | Meliphagidae | *Melithreptus* | *lunatus* |  |  |  | 5.7 |
| 16 | Passeriformes | Thamnophilidae | *Myrmotherula* | *brachyura* | 0.048 | 6.75 | 9.86 | 5.1 |
|  | Passeriformes | Thamnophilidae | *Myrmotherula* | *ignota* |  |  |  | 5.1 |
| 17 | Passeriformes | Thamnophilidae | *Myrmotherula* | *cherriei* | 0.02 | 8.25 | 1.2 | 5.1 |
|  | Passeriformes | Thamnophilidae | *Myrmotherula* | *surinamensis* |  |  |  | 5.1 |
| 18 | Passeriformes | Turdidae | *Neocossyphus* | *poensis* | 0.102 | 56.5 | 21.4 | 4.3 |
|  | Passeriformes | Turdidae | *Neocossyphus* | *rufus* |  |  |  | 4.3 |
| 19 | Passeriformes | Parulidae | *Oporornis* | *agilis* | 0.053 | 12.27 | 11.65 | 3.5 |
|  | Passeriformes | Parulidae | *Oporornis* | *philadelphia* |  |  |  | 3.5 |
| 20 | Passeriformes | Troglodytidae | *Pheugopedius* | *coraya* | 0.097 | 18.2 | 10.42 | 3.9 |
|  | Passeriformes | Troglodytidae | *Pheugopedius* | *genibarbis* |  |  |  | 3.9 |
| 21 | Piciformes | Picidae | *Picoides* | *pubescens* | 0.09 | 24.4 | 25.84 | 4.7 |
|  | Piciformes | Picidae | *Picoides* | *minor* |  |  |  | 5.2 |
| 22 | Passeriformes | Paridae | *Poecile* | *montanus* | 0.076 | 11.25 | 2.63 | 4.6 |
|  | Passeriformes | Paridae | *Poecile* | *palustris* |  |  |  | 4.2 |
| 23 | Passeriformes | Regulidae | *Regulus* | *ignicapillus* | 0.133 | 5.57 | 0.89 | 2.7 |
|  | Passeriformes | Regulidae | *Regulus* | *regulus* |  |  |  | 2.7 |
| 24 | Passeriformes | Parulidae | *Seiurus* | *motacilla* | 0.056 | 18.1 | 18.09 | 4.5 |
|  | Passeriformes | Parulidae | *Seiurus* | *novaboracens* |  |  |  | 4.4 |
| 25 | Passeriformes | Icteridae | *Sturnella* | *magna* | 0.044 | 65.25 | 53.37 | 4.4 |
|  | Passeriformes | Icteridae | *Sturnella* | *neglecta* |  |  |  | 4.2 |
| 26 | Passeriformes | Sylviidae | *Sylvia* | *atricapilla* | 0.134 | 17.45 | 8.24 | 4 |
|  | Passeriformes | Sylviidae | *Sylvia* | *borin* |  |  |  | 4 |
| 27 | Musophagiformes | Musophagidae | *Tauraco* | *macrorhynchus* | 0.168 | 265 | 20.1 | 3.3 |
|  | Musophagiformes | Musophagidae | *Tauraco* | *persa* |  |  |  | 3.3 |
| 28 | Passeriformes | Thamnophilidae | *Thamnomanes* | *ardesiacus* | 0.058 | 19.05 | 13.24 | 5.4 |
|  | Passeriformes | Thamnophilidae | *Thamnomanes* | *saturninus* |  |  |  | 5.3 |
| 29 | Passeriformes | Thamnophilidae | *Thamnomanes* | *caesius* | 0.058 | 16.45 | 8.72 | 4.9 |
|  | Passeriformes | Thamnophilidae | *Thamnomanes* | *schistogynus* |  |  |  | 5.7 |
| 30 | Passeriformes | Thamnophilidae | *Thamnophilus* | *murinus* | 0.088 | 19.8 | 4.93 | 4.9 |
|  | Passeriformes | Thamnophilidae | *Thamnophilus* | *schistaceus* |  |  |  | 4.9 |
| 31 | Passeriformes | Mimidae | *Toxostoma* | *bendirei* | 0.077 | 76.03 | 22.86 | 4.2 |
|  | Passeriformes | Mimidae | *Toxostoma* | *curvirostre* |  |  |  | 4.2 |
| 32 | Passeriformes | Parulidae | *Vermivora* | *chrysoptera* | 0.028 | 8.8 | 1.69 | 3.8 |
|  | Passeriformes | Parulidae | *Vermivora* | *pinus* |  |  |  | 3.7 |
| 33 | Passeriformes | Ptilonorhynchidae | *Amblyornis* | *inornatus* | 0.053 | 119.67 | 5.24 | 9.2 |
|  | Passeriformes | Ptilonorhynchidae | *Amblyornis* | *macgregoriae* |  |  |  | 9.2 |
| 34 | Passeriformes | Sylviidae | *Abroscopus* | *schisticeps* | 0.127 | 5.6 | 27.69 | 3.6 |
|  | Passeriformes | Sylviidae | *Abroscopus* | *superciliaris* |  |  |  | 3.6 |
| 35 | Passeriformes | Acanthizidae | *Acanthiza* | *nana* | 0.058 | 7.33 | 25.6 | 5.7 |
|  | Passeriformes | Acanthizidae | *Acanthiza* | *murina* |  |  |  | 5.7 |
| 36 | Passeriformes | Acanthizidae | *Acanthiza* | *uropygialis* | 0.062 | 6.22 | 5.78 | 5.7 |
|  | Passeriformes | Acanthizidae | *Acanthiza* | *iredalei* |  |  |  | 5.7 |
| 37 | Passeriformes | Acanthizidae | *Acanthiza* | *inornata* | 0.064 | 7.35 | 9.09 | 5.7 |
|  | Passeriformes | Acanthizidae | *Acanthiza* | *reguloides* |  |  |  | 5.7 |
| 38 | Passeriformes | Acanthizidae | *Acanthiza* | *katherina* | 0.043 | 7.58 | 6.49 | 5.7 |
|  | Passeriformes | Acanthizidae | *Acanthiza* | *pusilla* |  |  |  | 5.7 |
| 39 | Passeriformes | Meliphagidae | *Acanthorhynchus* | *superciliosus* | 0.122 | 10.01 | 5.02 | 5.6 |
|  | Passeriformes | Meliphagidae | *Acanthorhynchus* | *tenuirostris* |  |  |  | 5.6 |
| 40 | Coraciiformes | Bucerotidae | *Aceros* | *cassidix* | 0.067 | 1975 | 32.63 | 19 |
|  | Coraciiformes | Bucerotidae | *Aceros* | *corrugatus* |  |  |  | 19 |
| 41 | Passeriformes | Sylviidae | *Acrocephalus* | *palustris* | 0.085 | 11.9 | 6.5 | 4.4 |
|  | Passeriformes | Sylviidae | *Acrocephalus* | *scirpaceus* |  |  |  | 4.3 |
| 42 | Passeriformes | Sylviidae | *Acrocephalus* | *tangorum* | 0.092 | 9.6 | 0 | 4.4 |
|  | Passeriformes | Sylviidae | *Acrocephalus* | *agricola* |  |  |  | 4.4 |
| 43 | Passeriformes | Sylviidae | *Acrocephalus* | *orientalis* | 0.065 | 27.75 | 15 | 4.2 |
|  | Passeriformes | Sylviidae | *Acrocephalus* | *arundinaceus* |  |  |  | 4.2 |
| 44 | Galliformes | Cracidae | *Ortalis* | *garrula* | 0.011 | 569.67 | 9.6 | 5.7 |
|  | Galliformes | Cracidae | *Ortalis* | *ruficauda* |  |  |  | 5.7 |
| 45 | Galliformes | Cracidae | *Ortalis* | *guttata* | 0.048 | 546.33 | 2 | 5.7 |
|  | Galliformes | Cracidae | *Ortalis* | *canicollis* |  |  |  | 5.7 |
| 46 | Galliformes | Cracidae | *Ortalis* | *leucogastra* | 0.014 | 531.25 | 11.28 | 5.7 |
|  | Galliformes | Cracidae | *Ortalis* | *vetula* |  |  |  | 5.7 |
| 47 | Galliformes | Cracidae | *Chamaepetes* | *unicolor* | 0.042 | 864.33 | 35.77 | 5.7 |
|  | Galliformes | Cracidae | *Chamaepetes* | *goudotii* |  |  |  | 5.7 |
| 48 | Passeriformes | Turdidae | *Zoothera* | *heinei* | 0.04 | 82.8 |  | 3 |
|  | Passeriformes | Turdidae | *Zoothera* | *talaseae* |  |  |  | 3 |
| 49 | Passeriformes | Sylviidae | *Sylvia* | *leucomelaena* | 0.101 | 12.7 | 17.34 | 4 |
|  | Passeriformes | Sylviidae | *Sylvia* | *curruca* |  |  |  | 4 |
| 50 | Passeriformes | Sylviidae | *Sylvia* | *deserticola* | 0.026 | 9.33 | 20.37 | 4 |
|  | Passeriformes | Sylviidae | *Sylvia* | *undata* |  |  |  | 4.1 |
| 51 | Pelicaniformes | Sulidae | *Morus* | *capensis* | 0.018 | 2545.67 | 11.1 | 20.2 |
|  | Pelicaniformes | Sulidae | *Morus* | *serrator* |  |  |  | 21.2 |
| 52 | Passeriformes | Timaliidae | *Alcippe* | *brunnea* | 0.126 | 17.5 |  | 3.8 |
|  | Passeriformes | Timaliidae | *Alcippe* | *dubia* |  |  |  | 3.8 |
| 53 | Passeriformes | Timaliidae | *Alcippe* | *poioicephala* | 0.058 | 16.9 | 27.54 | 3.8 |
|  | Passeriformes | Timaliidae | *Alcippe* | *(peracensis) grotei* |  |  |  | 3.8 |
| 54 | Passeriformes | Hirundinidae | *Progne* | *chalybea* | 0.054 | 40.98 | 6.49 | 5.5 |
|  | Passeriformes | Hirundinidae | *Progne* | *dominicensis* |  |  |  | 5.5 |
| 55 | Gaviiformes | Procellariidae | *Pseudobulweria* | *aterrima* | 0.057 | 320.5 | 43.28 | 17.7 |
|  | Gaviiformes | Procellariidae | *Pseudobulweria* | *rostrata* |  |  |  | 17.7 |
| 56 | Passeriformes | Hirundinidae | *Delichon* | *dasypus* | 0.049 | 16.5 | 16.67 | 4.3 |
|  | Passeriformes | Hirundinidae | *Delichon* | *nipalensis* |  |  |  | 4.3 |
| 57 | Passeriformes | Fringillidae | *Carduelis* | *spinus* | 0.022 | 13.07 | 4.15 | 4.1 |
|  | Passeriformes | Fringillidae | *Carduelis* | *pinus* |  |  |  | 3.9 |
| 58 | Passeriformes | Fringillidae | *Serinus* | *flaviventris* | 0.026 | 22.05 | 41.94 | 3.8 |
|  | Passeriformes | Fringillidae | *Serinus* | *sulphuratus* |  |  |  | 3.5 |
| 59 | Passeriformes | Thraupidae | *Iridosornis* | *porphyrocephalus* | 0.06 | 26 |  | 3.7 |
|  | Passeriformes | Thraupidae | *Iridosornis* | *analis* |  |  |  | 3.7 |
| 60 | Piciformes | Picidae | *Sasia* | *abnormis* | 0.119 | 9.45 | 5.15 | 3.8 |
|  | Piciformes | Picidae | *Sasia* | *ochracea* |  |  |  | 3.8 |
| 61 | Trogoniiformes | Trogonidae | *Pharomachrus* | *antisianus* | 0.059 | 156.33 | 6.13 | 7.3 |
|  | Trogoniiformes | Trogonidae | *Pharomachrus* | *pavoninus* |  |  |  | 7.3 |
| 62 | Galliformes | Phasianidae | *Lagopus* | *muta* | 0.056 | 540.89 | 1.97 | 4.2 |
|  | Galliformes | Phasianidae | *Lagopus* | *lagopus* |  |  |  | 4.2 |
| 63 | Passeriformes | Emberizidae | *Emberiza* | *citrinella* | 0.002 | 29.1 | 4.04 | 3.7 |
|  | Passeriformes | Emberizidae | *Emberiza* | *leucocephalos* |  |  |  | 3.6 |
| 64 | Passeriformes | Emberizidae | *Emberiza* | *pallasi* | 0.059 | 16.23 | 24.12 | 3.6 |
|  | Passeriformes | Emberizidae | *Emberiza* | *schoeniclus* |  |  |  | 3.6 |
| 65 | Passeriformes | Emberizidae | *Emberiza* | *hortulana* | 0.01 | 19.53 | 3.77 | 3.6 |
|  | Passeriformes | Emberizidae | *Emberiza* | *caesia* |  |  |  | 3.6 |
| 66 | Passeriformes | Emberizidae | *Emberiza* | *striolata* | 0.078 | 14.25 | 7.43 | 3.6 |
|  | Passeriformes | Emberizidae | *Emberiza* | *tahapisi* |  |  |  | 3.6 |
| 67 | Charadriiformes | Alcidae | *Brachyramphus* | *marmoratus* | 0.063 | 227 | 5.98 | 12.1 |
|  | Charadriiformes | Alcidae | *Brachyramphus* | *brevirostris* |  |  |  | 12.1 |
| 68 | Piciformes | Ramphastidae | *Pteroglossus* | *castanotis* | 0.021 | 260.93 | 1.8 | 7 |
|  | Piciformes | Ramphastidae | *Pteroglossus* | *pluricinctus* |  |  |  | 7 |
| 69 | Passeriformes | Paradisaeidae | *Cicinnurus* | *magnificus* | 0.061 | 74.03 | 34.68 | 7.9 |
|  | Passeriformes | Paradisaeidae | *Cicinnurus* | *respublica* |  |  |  | 7.9 |
| 70 | Falconiformes | Accipitridae | *Spizaetus* | *ornatus* | 0.06 | 1215 |  | 18.5 |
|  | Falconiformes | Accipitridae | *Spizaetus (Oroaetus)* | *isidori* |  |  |  | 18.5 |
| 71 | Passeriformes | Uncertain | *Granatellus* | *venustus* | 0.078 | 10.35 | 8.33 | 3.9 |
|  | Passeriformes | Uncertain | *Granatellus* | *sallaei* |  |  |  | 3.9 |
| 72 | Charadriiformes | Alcidae | *Fratercula* | *arctica* | 0.03 | 484.67 | 28.98 | 21.6 |
|  | Charadriiformes | Alcidae | *Fratercula* | *corniculata* |  |  |  | 21.6 |
| 73 | Psittaciformes | Psittacidae | *Amazona* | *viridigenalis* | 0.035 | 309 | 4.43 | 12.3 |
|  | Psittaciformes | Psittacidae | *Amazona* | *finschi* |  |  |  | 12.3 |
| 74 | Psittaciformes | Psittacidae | *Amazona* | *guildingii* | 0.063 | 508 | 42.72 | 12.3 |
|  | Psittaciformes | Psittacidae | *Amazona* | *amazonica* |  |  |  | 12.3 |
| 75 | Passeriformes | Bombycillidae | *Bombycilla* | *garrulus* | 0.056 | 48.3 | 22.09 | 4.6 |
|  | Passeriformes | Bombycillidae | *Bombycilla* | *japonica* |  |  |  | 4.4 |
| 76 | Anseriformes | Anatidae | *Aix* | *sponsa* | 0.082 | 517.25 | 18.51 | 7.4 |
|  | Anseriformes | Anatidae | *Aix* | *galericulata* |  |  |  | 7.4 |
| 77 | Charadriiformes | Laridae | *Rissa* | *tridactyla* | 0.044 | 397.33 | 7.48 | 12.9 |
|  | Charadriiformes | Laridae | *Rissa* | *brevirostris* |  |  |  | 12.9 |
| 78 | Ciconiiformes | Ciconiidae | *Mycteria* | *leucocephala* | 0.01 | 3180 |  | 8.4 |
|  | Ciconiiformes | Ciconiidae | *Mycteria* | *cinerea* |  |  |  | 8.4 |
| 79 | Galliformes | Phasianidae | *Falcipennis* | *falcipennis* | 0.029 | 580.75 | 31.05 | 5 |
|  | Galliformes | Phasianidae | *Falcipennis* | *canadensis* |  |  |  | 5 |
| 80 | Passeriformes | Emberizidae | *Emberiza* | *bruniceps* | 0.052 | 27.17 | 12.52 | 3.6 |
|  | Passeriformes | Emberizidae | *Emberiza* | *melanocephala* |  |  |  | 3.6 |
| 81 | Charadriiformes | Scolopacidae | *Tringa* | *melanoleuca* | 0.091 | 174.89 | 12.96 | 5.7 |
|  | Charadriiformes | Scolopacidae | *Tringa* | *nebularia* |  |  |  | 6.3 |
| 82 | Charadriiformes | Scolopacidae | *Tringa* | *flavipes* | 0.088 | 193.13 | 69.12 | 5.7 |
|  | Charadriiformes | Scolopacidae | *Tringa* | *semipalmatus* |  |  |  | 7.5 |
| 83 | Gaviiformes | Gaviidae | *Gavia* | *arctica* | 0.056 | 2374 | 45.82 | 9.8 |
|  | Gaviiformes | Gaviidae | *Gavia* | *pacifica* |  |  |  | 9.8 |
| 84 | Passeriformes | Sylviidae | *Phylloscopus* | *inornatus* | 0.082 | 6.26 | 7.98 | 3.6 |
|  | Passeriformes | Sylviidae | *Phylloscopus* | *humei* |  |  |  | 3.6 |
| 85 | Passeriformes | Turdidae | *Catharus* | *minimus* | 0.01 | 29.7 | 12.03 | 4 |
|  | Passeriformes | Turdidae | *Catharus* | *bicknelli* |  |  |  | 4.2 |
| 86 | Passeriformes | Sylviidae | *Hippolais* | *caligata* | 0.075 | 9.1 | 4.3 | 4 |
|  | Passeriformes | Sylviidae | *Hippolais* | *rama* |  |  |  | 4 |
| 87 | Passeriformes | Sylviidae | *Phylloscopus* | *bonelli* | 0.086 | 8.63 | 2.3 | 3.6 |
|  | Passeriformes | Sylviidae | *Phylloscopus* | *orientalis* |  |  |  | 3.6 |
| 88 | Anseriformes | Anatidae | *Anas* | *americana* | 0.005 | 844.5 | 19.07 | 7 |
|  | Anseriformes | Anatidae | *Anas* | *sibilatrix* |  |  |  | 6.6 |
| 89 | Anseriformes | Anatidae | *Anas* | *crecca* | 0.06 | 323.25 | 10.41 | 6.3 |
|  | Anseriformes | Anatidae | *Anas* | *carolinensis* |  |  |  | 6.3 |
| 90 | Anseriformes | Anatidae | *Aythya* | *marila* | 0.017 | 913.25 | 18.53 | 8.2 |
|  | Anseriformes | Anatidae | *Aythya* | *affinis* |  |  |  | 7.7 |
| 91 | Anseriformes | Anatidae | *Somateria* | *spectabilis* | 0.004 | 1842 | 21.73 | 9 |
|  | Anseriformes | Anatidae | *Somateria* | *mollissima* |  |  |  | 9 |
| 92 | Anseriformes | Anatidae | *Bucephala* | *clangula* | 0.033 | 931.64 | 1.88 | 8 |
|  | Anseriformes | Anatidae | *Bucephala* | *islandica* |  |  |  | 8.1 |
| 93 | Charadriiformes | Scolopacidae | *Actitis* | *hypoleucos* | 0.115 | 44.2 | 15.83 | 6.8 |
|  | Charadriiformes | Scolopacidae | *Actitis* | *macularia* |  |  |  | 5.8 |
| 94 | Falconiformes | Accipitridae | *Gyps* | *fulvus* | 0.008 | 7418 | 0.48 | 17.8 |
|  | Falconiformes | Accipitridae | *Gyps* | *rueppellii* |  |  |  | 18.8 |
| 95 | Psittaciformes | Psittacidae | *Eunymphicus* | *cornatus* | 0.008 | 130 | 0 | 6.3 |
|  | Psittaciformes | Psittacidae | *Eunymphicus* | *uvaeensis* |  |  |  | 6.3 |
| 96 | Coliiformes | Coliidae | *Colius* | *striatus* | 0.104 | 46.25 | 18.98 | 2.7 |
|  | Coliiformes | Coliidae | *Colius* | *colius* |  |  |  | 2.2 |
| 97 | Phoenicopteriformes | Phoenicopteridae | *Phoenicopterus* | *roseus* | 0.016 | 3065.75 | 1.99 | 16.3 |
|  | Phoenicopteriformes | Phoenicopteridae | *Phoenicopterus* | *ruber* |  |  |  | 15 |
| 98 | Gaviiformes | Gaviidae | *Gavia* | *immer* | 0.007 | 5018.13 | 1.51 | 9.8 |
|  | Gaviiformes | Gaviidae | *Gavia* | *adamsii* |  |  |  | 9.8 |
| 99 | Caprimulgiformes | Caprimulgidae | *Caprimulgus* | *fossii* | 0.053 | 54.13 | 21.44 | 5.6 |
|  | Caprimulgiformes | Caprimulgidae | *Caprimulgus* | *climacurus* |  |  |  | 5.6 |
| 100 | Caprimulgiformes | Caprimulgidae | *Caprimulgus* | *pectoralis* | 0 | 47.15 | 20.38 | 5.6 |
|  | Caprimulgiformes | Caprimulgidae | *Caprimulgus* | *poliocephalus* |  |  |  | 5.6 |
| 101 | Caprimulgiformes | Caprimulgidae | *Caprimulgus* | *affinis* | 0.081 | 68 | 24.06 | 5.6 |
|  | Caprimulgiformes | Caprimulgidae | *Caprimulgus* | *aegyptius* |  |  |  | 5.6 |
| 102 | Caprimulgiformes | Caprimulgidae | *Caprimulgus* | *carolinensis* | 0.054 | 101.6 | 13.58 | 5.6 |
|  | Caprimulgiformes | Caprimulgidae | *Caprimulgus* | *rufus* |  |  |  | 5.6 |
| 103 | Caprimulgiformes | Caprimulgidae | *Caprimulgus* | *saturatus* | 0.064 | 52.93 | 2.62 | 5.6 |
|  | Caprimulgiformes | Caprimulgidae | *Caprimulgus* | *vociferus* |  |  |  | 5.6 |
| 104 | Caprimulgiformes | Caprimulgidae | *Caprimulgus* | *cayennensis* | 0.086 | 32.9 | 14.65 | 5.6 |
|  | Caprimulgiformes | Caprimulgidae | *Caprimulgus* | *maculicaudus* |  |  |  | 5.6 |
| 105 | Caprimulgiformes | Caprimulgidae | *Chordeiles* | *rupestris* | 0.069 | 49.9 |  | 5 |
|  | Caprimulgiformes | Caprimulgidae | *Chordeiles* | *acutipennis* |  |  |  | 5 |
| 106 | Caprimulgiformes | Caprimulgidae | *Nyctiphrynus* | *yucatanicus* | 0.089 | 32.05 | 38.13 | 5.4 |
|  | Caprimulgiformes | Caprimulgidae | *Nyctiphrynus* | *ocellatus* |  |  |  | 5.4 |
| 107 | Caprimulgiformes | Caprimulgidae | *Lurocalis* | *semitorquatus* | 0.061 | 75.9 |  | 5.4 |
|  | Caprimulgiformes | Caprimulgidae | *Lurocalis* | *rufiventris* |  |  |  | 5.4 |
| 108 | Caprimulgiformes | Caprimulgidae | *Macrodipteryx* | *longipennis* | 0.066 | 64.27 | 33.7 | 5.4 |
|  | Caprimulgiformes | Caprimulgidae | *Macrodipteryx* | *vexillarius* |  |  |  | 5.4 |
| 109 | Caprimulgiformes | Caprimulgidae | *Hydropsalis* | *climacocerca* | 0.059 | 53.87 | 20.42 | 5.4 |
|  | Caprimulgiformes | Caprimulgidae | *Hydropsalis* | *torquata* |  |  |  | 5.4 |
| 110 | Caprimulgiformes | Caprimulgidae | *Uropsalis* | *segmentata* | 0.046 | 57.05 | 39.75 | 5.4 |
|  | Caprimulgiformes | Caprimulgidae | *Uropsalis* | *lyra* |  |  |  | 5.4 |
| 111 | Caprimulgiformes | Aegothelidae | *Aegotheles* | *archboldi* | 0.05 | 32.9 | 13.07 | 5.4 |
|  | Caprimulgiformes | Aegothelidae | *Aegotheles* | *albertisi* |  |  |  | 5.4 |
| 112 | Caprimulgiformes | Aegothelidae | *Aegotheles* | *bennettii* | 0.064 | 50.29 | 18.64 | 5.4 |
|  | Caprimulgiformes | Aegothelidae | *Aegotheles* | *cristatus* |  |  |  | 5.4 |
| 113 | Caprimulgiformes | Nyctibiidae | *Nyctibius* | *grandis* | 0.179 | 493.75 | 19.47 | 7.5 |
|  | Caprimulgiformes | Nyctibiidae | *Nyctibius* | *aethereus* |  |  |  | 7.5 |
| 114 | Caprimulgiformes | Nyctibiidae | *Nyctibius* | *maculosus* | 0.127 | 129.45 | 49.91 | 7.5 |
|  | Caprimulgiformes | Nyctibiidae | *Nyctibius* | *leucopterus* |  |  |  | 7.5 |
| 115 | Caprimulgiformes | Nyctibiidae | *Nyctibius* | *griseus* | 0.027 | 229.98 | 31.02 | 7.5 |
|  | Caprimulgiformes | Nyctibiidae | *Nyctibius* | *jamaicensis* |  |  |  | 7.5 |
| 116 | Caprimulgiformes | Podargidae | *Podargus* | *papuensis* | 0.05 | 336.8 | 17.97 | 7.5 |
|  | Caprimulgiformes | Podargidae | *Podargus* | *strigoides* |  |  |  | 7.5 |
| 117 | Caprimulgiformes | Caprimulgidae | *Eurostopodus* | *argus* | 0.045 | 123.8 | 32.81 | 5.4 |
|  | Caprimulgiformes | Caprimulgidae | *Eurostopodus* | *mystacalis* |  |  |  | 5.4 |
| 118 | Apodiformes | Trochilidae | *Lampornis* | *viridipallens* | 0.053 | 5.35 |  | 4.5 |
|  | Apodiformes | Trochilidae | *Lampornis* | *sybillae* |  |  |  | 4.5 |
| 119 | Apodiformes | Trochilidae | *Lampornis* | *clemenciae* | 0.124 | 6.9 | 27.63 | 4.5 |
|  | Apodiformes | Trochilidae | *Lampornis* | *hemileucus* |  |  |  | 4.5 |
| 120 | Apodiformes | Trochilidae | *Metallura* | *theresiae* | 0.003 | 4.75 | 6.12 | 4.2 |
|  | Apodiformes | Trochilidae | *Metallura* | *eupogon* |  |  |  | 4.2 |
| 121 | Apodiformes | Trochilidae | *Metallura* | *williami* | 0.003 | 4.4 | 4.44 | 4.2 |
|  | Apodiformes | Trochilidae | *Metallura* | *baroni* |  |  |  | 4.2 |
| 122 | Apodiformes | Trochilidae | *Sephanoides* | *sephanoides* | 0.052 | 7.08 | 41.9 | 4.2 |
|  | Apodiformes | Trochilidae | *Sephanoides* | *fernandensis* |  |  |  | 4.2 |
| 123 | Apodiformes | Trochilidae | *Basilinna* | *xantusii* | 0.059 | 3.4 | 0 | 4.2 |
|  | Apodiformes | Trochilidae | *Basilinna* | *leucotis* |  |  |  | 4.2 |
| 124 | Ciconiiformes | Threskiornithidae | *Platalea* | *leucorodia* | 0.028 | 1548 | 34.26 | 7.2 |
|  | Ciconiiformes | Threskiornithidae | *Platalea* | *minor* |  |  |  | 7.2 |
| 125 | Ciconiiformes | Threskiornithidae | *Plegadis* | *falcinellus* | 0.033 | 608.88 | 7.85 | 6.7 |
|  | Ciconiiformes | Threskiornithidae | *Plegadis* | *ridgwayi* |  |  |  | 6.7 |
| 126 | Ciconiiformes | Ardeidae | *Botaurus* | *stellaris* | 0.1 | 1118.33 | 46.7 | 5.5 |
|  | Ciconiiformes | Ardeidae | *Botaurus* | *lentiginosus* |  |  |  | 5.5 |
| 127 | Ciconiiformes | Ardeidae | *Tigrisoma* | *lineatum* | 0.084 | 831.5 | 4.35 | 7.4 |
|  | Ciconiiformes | Ardeidae | *Tigrisoma* | *fasciatum* |  |  |  | 7.4 |
| 128 | Ciconiiformes | Ciconiidae | *Ciconia* | *ciconia* | 0.036 | 4149.25 | 28.91 | 16.5 |
|  | Ciconiiformes | Ciconiidae | *Ciconia* | *boyciana* |  |  |  | 16.1 |
| 129 | Ciconiiformes | Ciconiidae | *Ciconia* | *episcopus* | 0.034 | 2061 |  | 15.9 |
|  | Ciconiiformes | Ciconiidae | *Ciconia* | *stormi* |  |  |  | 16.1 |
| 130 | Ciconiiformes | Ciconiidae | *Ephippiorhynchus* | *asiaticus* | 0.085 | 5475 | 33.47 | 16.1 |
|  | Ciconiiformes | Ciconiidae | *Ephippiorhynchus* | *senegalensis* |  |  |  | 16.1 |
| 131 | Falconiformes | Cathartidae | *Cathartes* | *burrovianus* | 0.042 | 1154 | 31.9 | 9.9 |
|  | Falconiformes | Cathartidae | *Cathartes* | *melambrotus* |  |  |  | 9.9 |
| 132 | Columbiformes | Columbidae | *Streptopelia* | *decaocto* | 0.023 | 151 | 3.87 | 5.3 |
|  | Columbiformes | Columbidae | *Streptopelia* | *roseogrisea* |  |  |  | 5.3 |
| 133 | Columbiformes | Columbidae | *Streptopelia* | *capicola* | 0.024 | 126 | 22.54 | 5.3 |
|  | Columbiformes | Columbidae | *Streptopelia* | *vinacea* |  |  |  | 5.3 |
| 134 | Columbiformes | Columbidae | *Streptopelia* | *turtur* | 0.013 | 147 | 18.52 | 5.3 |
|  | Columbiformes | Columbidae | *Streptopelia* | *hypopyrrha* |  |  |  | 5.3 |
| 135 | Columbiformes | Columbidae | *Streptopelia* | *chinensis* | 0.082 | 129.97 | 36.52 |  |
|  | Columbiformes | Columbidae | *Streptopelia* | *senegalensis* |  |  |  | 4 |
| 136 | Columbiformes | Columbidae | *Streptopelia* | *mayeri* | 0.036 | 227.4 | 41.58 | 4 |
|  | Columbiformes | Columbidae | *Streptopelia* | *picturata* |  |  |  | 5.6 |
| 137 | Columbiformes | Columbidae | *Zenaida* | *macroura* | 0.01 | 143.33 | 38.02 | 6.7 |
|  | Columbiformes | Columbidae | *Zenaida* | *graysoni* |  |  |  | 6.8 |
| 138 | Columbiformes | Columbidae | *Zenaida* | *asiatica* | 0.045 | 153 |  | 7.1 |
|  | Columbiformes | Columbidae | *Zenaida* | *meloda* |  |  |  | 6.8 |
| 139 | Columbiformes | Columbidae | *Zenaida* | *auriculata* | 0.042 | 115.91 | 18.78 | 6.8 |
|  | Columbiformes | Columbidae | *Zenaida* | *galapagoensis* |  |  |  | 6.8 |
| 140 | Columbiformes | Columbidae | *Hemiphaga* | *novaseelandiae* | 0.01 | 653 |  | 6.6 |
|  | Columbiformes | Columbidae | *Hemiphaga* | *chathamensis* |  |  |  | 6.6 |
| 141 | Columbiformes | Columbidae | *Leptotila* | *megalura* | 0.047 | 187.5 | 27.98 | 4.2 |
|  | Columbiformes | Columbidae | *Leptotila* | *rufaxilla* |  |  |  | 4.2 |
| 142 | Columbiformes | Columbidae | *Geotrygon* | *albifacies* | 0.062 | 273 | 22.73 | 4.6 |
|  | Columbiformes | Columbidae | *Geotrygon* | *chiriquensis* |  |  |  | 4.6 |
| 143 | Columbiformes | Columbidae | *Columbina* | *picui* | 0.073 | 48.55 | 6.19 | 4.3 |
|  | Columbiformes | Columbidae | *Columbina* | *cruziana* |  |  |  | 4.3 |
| 144 | Columbiformes | Columbidae | *Columbina* | *minuta* | 0.015 | 39.15 | 29.58 | 4.3 |
|  | Columbiformes | Columbidae | *Columbina* | *talpacoti* |  |  |  | 4.3 |
| 145 | Columbiformes | Columbidae | *Turtur* | *chalcospilos* | 0.08 | 63.1 | 7.62 | 8.5 |
|  | Columbiformes | Columbidae | *Turtur* | *afer* |  |  |  | 8.5 |
| 146 | Columbiformes | Columbidae | *Chalcophaps* | *indica* | 0.089 | 132.4 | 13.47 | 5.6 |
|  | Columbiformes | Columbidae | *Chalcophaps* | *stephani* |  |  |  | 5.6 |
| 147 | Columbiformes | Columbidae | *Goura* | *cristata* | 0.071 | 2192 | 16.11 | 6.6 |
|  | Columbiformes | Columbidae | *Goura* | *victoria* |  |  |  | 6.6 |
| 148 | Columbiformes | Columbidae | *Metriopelia* | *ceciliae* | 0.039 | 55.1 |  | 4.6 |
|  | Columbiformes | Columbidae | *Metriopelia* | *morenoi* |  |  |  | 4.6 |
| 149 | Gruiformes | Otididae | *Chlamydotis* | *undulata* | 0.019 | 1555 |  | 10.3 |
|  | Gruiformes | Otididae | *Chlamydotis* | *macqueenii* |  |  |  | 10.3 |
| 150 | Gruiformes | Otididae | *Ardeotis* | *arabs* | 0.047 | 7317.5 | 27.01 | 15.6 |
|  | Gruiformes | Otididae | *Ardeotis* | *kori* |  |  |  | 15.6 |
| 151 | Gruiformes | Gruidae | *Grus* | *monacha* | 0.015 | 4490 | 37.75 | 13 |
|  | Gruiformes | Gruidae | *Grus* | *nigricollis* |  |  |  | 13 |
| 152 | Gruiformes | Gruidae | *Grus* | *vipio* | 0.033 | 5721.33 | 25.4 | 13 |
|  | Gruiformes | Gruidae | *Grus* | *rubicunda* |  |  |  | 12.5 |
| 153 | Gruiformes | Gruidae | *Anthropoides* | *virgo* | 0.032 | 3908.33 | 48.07 | 11.2 |
|  | Gruiformes | Gruidae | *Anthropoides* | *paradiseus* |  |  |  | 13 |
| 154 | Gruiformes | Gruidae | *Balearica* | *regulorum* | 0.035 | 3681 | 4.83 | 15.1 |
|  | Gruiformes | Gruidae | *Balearica* | *pavonina* |  |  |  | 15.1 |
| 155 | Gruiformes | Rallidae | *Porphyrio* | *porphyrio* | 0.043 | 924.8 |  | 5.9 |
|  | Gruiformes | Rallidae | *Porphyrio* | *hochstetteri* |  |  |  | 9.8 |
| 156 | Gruiformes | Psophiidae | *Psophia* | *crepitans* | 0.035 | 1048.5 | 4.2 | 10.4 |
|  | Gruiformes | Psophiidae | *Psophia* | *viridis* |  |  |  | 10.4 |
| 157 | Pelicaniformes | Sulidae | *Sula* | *dactylatra* | 0.015 | 1748.58 | 0.62 | 16.3 |
|  | Pelicaniformes | Sulidae | *Sula* | *granti* |  |  |  | 10 |
| 158 | Pelicaniformes | Sulidae | *Sula* | *nebouxii* | 0.009 | 1488.5 | 6.94 | 8.5 |
|  | Pelicaniformes | Sulidae | *Sula* | *variegata* |  |  |  | 10.6 |
| 159 | Pelicaniformes | Pelecanidae | *Pelecanus* | *erythrorhynchos* | 0.083 | 4543.75 | 39.15 | 15 |
|  | Pelicaniformes | Pelecanidae | *Pelecanus* | *occidentalis* |  |  |  | 15 |
| 160 | Apodiformes | Apodidae | *Aerodramus* | *bartschi* | 0.003 | 8 | 13.95 | 5 |
|  | Apodiformes | Apodidae | *Aerodramus* | *sawtelli* |  |  |  | 5 |
| 161 | Apodiformes | Apodidae | *Aerodramus* | *elaphrus* | 0.016 | 9.8 | 16.82 | 5 |
|  | Apodiformes | Apodidae | *Aerodramus* | *francicus* |  |  |  | 5 |
| 162 | Apodiformes | Apodidae | *Aerodramus* | *brevirostris* | 0.033 | 14.8 | 25.88 | 5 |
|  | Apodiformes | Apodidae | *Aerodramus* | *maximus* |  |  |  | 5 |
| 163 | Apodiformes | Apodidae | *Cypsiurus* | *parvus* | 0.065 | 11.38 | 32.72 | 7.2 |
|  | Apodiformes | Apodidae | *Cypsiurus* | *balasiensis* |  |  |  | 7.2 |
| 164 | Anseriformes | Anatidae | *Anas* | *strepera* | 0.018 | 783 | 29.23 | 7.1 |
|  | Anseriformes | Anatidae | *Anas* | *falcata* |  |  |  | 6.6 |
| 165 | Anseriformes | Anatidae | *Anas* | *georgica* | 0.019 | 777.29 | 35.76 | 6.6 |
|  | Anseriformes | Anatidae | *Anas* | *acuta* |  |  |  | 6.8 |
| 166 | Anseriformes | Anatidae | *Anas* | *gracilis* | 0 | 557.29 | 21.67 | 6.6 |
|  | Anseriformes | Anatidae | *Anas* | *castanea* |  |  |  | 6.6 |
| 167 | Anseriformes | Anatidae | *Anas* | *versicolor* | 0.009 | 596 | 10.21 | 6.6 |
|  | Anseriformes | Anatidae | *Anas* | *puna* |  |  |  | 6.6 |
| 168 | Anseriformes | Anatidae | *Anas* | *discors* | 0.003 | 368.75 | 4.64 | 6.4 |
|  | Anseriformes | Anatidae | *Anas* | *cyanoptera* |  |  |  | 6.5 |
| 169 | Anseriformes | Anatidae | *Anas* | *rhynchotis* | 0.002 | 628.8 | 4.12 | 6.6 |
|  | Anseriformes | Anatidae | *Anas* | *clypeata* |  |  |  | 6.5 |
| 170 | Anseriformes | Anatidae | *Anas* | *laysanensis* | 0.011 | 747.25 | 55.6 | 6.5 |
|  | Anseriformes | Anatidae | *Anas* | *superciliosa* |  |  |  | 6.6 |
| 171 | Anseriformes | Anatidae | *Anser* | *albifrons* | 0.004 | 2024.29 | 23.02 | 11.3 |
|  | Anseriformes | Anatidae | *Anser* | *erythropus* |  |  |  | 11.4 |
| 172 | Anseriformes | Anatidae | *Branta* | *leucopsis* | 0.068 | 1460.75 | 26.82 | 10.5 |
|  | Anseriformes | Anatidae | *Branta* | *ruficollis* |  |  |  | 10.9 |
| 173 | Anseriformes | Anatidae | *Cygnus* | *atratus* | 0.102 | 5212.5 | 15.49 | 12.6 |
|  | Anseriformes | Anatidae | *Cygnus* | *melanocoryphus* |  |  |  | 12.6 |
| 174 | Anseriformes | Anatidae | *Oxyura* | *maccoa* | 0.058 | 676 | 3.2 | 6 |
|  | Anseriformes | Anatidae | *Oxyura* | *leucocephala* |  |  |  | 6 |
| 175 | Anseriformes | Anatidae | *Tadorna* | *ferruginea* | 0.002 | 1221.67 | 4.79 | 10.9 |
|  | Anseriformes | Anatidae | *Tadorna* | *cana* |  |  |  | 10.9 |
| 176 | Anseriformes | Anatidae | *Tadorna* | *tadorna* | 0.091 | 1019.25 | 23.05 | 10.9 |
|  | Anseriformes | Anatidae | *Tadorna* | *radjah* |  |  |  | 10.9 |
| 177 | Anseriformes | Anatidae | *Dendrocygna* | *arcuata* | 0.077 | 707.67 | 34.29 | 5.3 |
|  | Anseriformes | Anatidae | *Dendrocygna* | *javanica* |  |  |  | 5.3 |
| 178 | Anseriformes | Anatidae | *Netta* | *rufina* | 0.047 | 1059 | 10.55 | 7 |
|  | Anseriformes | Anatidae | *Netta* | *peposaca* |  |  |  | 7 |
| 179 | Anseriformes | Anatidae | *Mergus* | *merganser* | 0.014 | 1246 | 30.53 | 7.3 |
|  | Anseriformes | Anatidae | *Mergus* | *serrator* |  |  |  | 7.3 |
| 180 | Strigiformes | Strigidae | *Asio* | *flammeus* | 0.082 | 341.81 | 3.39 | 7.2 |
|  | Strigiformes | Strigidae | *Asio* | *capensis* |  |  |  | 7.2 |
| 181 | Strigiformes | Strigidae | *Athene* | *brama* | 0.135 | 141 | 25.66 | 4 |
|  | Strigiformes | Strigidae | *Athene* | *cunicularia* |  |  |  | 4.6 |
| 182 | Strigiformes | Strigidae | *Pulsatrix* | *perspicillata* | 0.103 | 740.67 | 44.74 | 5.7 |
|  | Strigiformes | Strigidae | *Pulsatrix* | *koeniswaldiana* |  |  |  | 5.7 |
| 183 | Strigiformes | Strigidae | *Ptilopsis* | *leucotis* | 0.018 | 197 | 6.86 | 3.7 |
|  | Strigiformes | Strigidae | *Ptilopsis* | *granti* |  |  |  | 3.7 |
| 184 | Strigiformes | Tytonidae | *Tyto* | *tenebricosa* | 0.004 | 576.5 | 32.78 | 6.1 |
|  | Strigiformes | Tytonidae | *Tyto* | *multipunctata* |  |  |  | 6.1 |
| 185 | Procellariiformes | Procellariidae | *Puffinus* | *pacificus* | 0.033 | 397.5 | 4.67 | 16.5 |
|  | Procellariiformes | Procellariidae | *Puffinus* | *bulleri* |  |  |  | 18.3 |
| 186 | Procellariiformes | Procellariidae | *Puffinus* | *creatopus* | 0.007 | 676.5 | 18.15 | 18.3 |
|  | Procellariiformes | Procellariidae | *Puffinus* | *carneipes* |  |  |  | 18.3 |
| 187 | Procellariiformes | Procellariidae | *Puffinus* | *griseus* | 0.037 | 818 | 7.3 | 21.2 |
|  | Procellariiformes | Procellariidae | *Puffinus* | *gravis* |  |  |  | 18.3 |
| 188 | Procellariiformes | Procellariidae | *Puffinus* | *gavia* | 0.045 | 274 | 37.09 | 18.3 |
|  | Procellariiformes | Procellariidae | *Puffinus* | *huttoni* |  |  |  | 19.5 |
| 189 | Procellariiformes | Procellariidae | *Pterodroma* | *magentae* | 0.021 | 492.5 | 10.58 | 19.5 |
|  | Procellariiformes | Procellariidae | *Pterodroma* | *incerta* |  |  |  | 15.6 |
| 190 | Procellariiformes | Procellariidae | *Pterodroma* | *macroptera* | 0.018 | 587.67 | 23.71 | 13.6 |
|  | Procellariiformes | Procellariidae | *Pterodroma* | *lessonii* |  |  |  | 12.6 |
| 191 | Procellariiformes | Procellariidae | *Pterodroma* | *cookii* | 0.059 | 166.67 | 19.89 | 15.6 |
|  | Procellariiformes | Procellariidae | *Pterodroma* | *longirostris* |  |  |  | 15.6 |
| 192 | Procellariiformes | Procellariidae | *Pterodroma* | *heraldica* | 0.007 | 330 | 32.49 | 15.6 |
|  | Procellariiformes | Procellariidae | *Pterodroma* | *arminjoniana* |  |  |  | 15.6 |
| 193 | Procellariiformes | Procellariidae | *Pterodroma* | *feae* | 0.007 | 311 |  | 14.5 |
|  | Procellariiformes | Procellariidae | *Pterodroma* | *deserta* |  |  |  | 14.5 |
| 194 | Procellariiformes | Procellariidae | *Calonectris* | *leucomelas* | 0.035 | 557.5 | 7.76 | 19.3 |
|  | Procellariiformes | Procellariidae | *Calonectris* | *diomedea* |  |  |  | 19.3 |
| 195 | Procellariiformes | Procellariidae | *Procellaria* | *cinerea* | 0.032 | 1172 | 6.76 | 25.2 |
|  | Procellariiformes | Procellariidae | *Procellaria* | *aequinoctialis* |  |  |  | 24.7 |
| 196 | Procellariiformes | Procellariidae | *Macronectes* | *giganteus* | 0.01 | 4477.6 | 14.04 | 21.3 |
|  | Procellariiformes | Procellariidae | *Macronectes* | *halli* |  |  |  | 17 |
| 197 | Procellariiformes | Hydrobatidae | *Oceanodroma* | *tristrami* | 0.09 | 63.88 | 59.14 | 14.6 |
|  | Procellariiformes | Hydrobatidae | *Oceanodroma* | *leucorhoa* |  |  |  | 13 |
| 198 | Procellariiformes | Procellariidae | *Fulmarus* | *glacialis* | 0.045 | 704 | 22.89 | 30.7 |
|  | Procellariiformes | Procellariidae | *Fulmarus* | *glacialoides* |  |  |  | 23.3 |
| 199 | Procellariiformes | Procellariidae | *Pachyptila* | *salvini* | 0.007 | 153 | 7.55 | 11.7 |
|  | Procellariiformes | Procellariidae | *Pachyptila* | *desolata* |  |  |  | 11.3 |
| 200 | Procellariiformes | Hydrobatidae | *Fregetta* | *grallaria* | 0.081 | 51.08 | 3.56 | 15.2 |
|  | Procellariiformes | Hydrobatidae | *Fregetta* | *tropica* |  |  |  | 15.2 |
| 201 | Procellariiformes | Diomedeidae | *Phoebastria* | *immutabilis* | 0.018 | 3172.5 | 1.41 | 28.5 |
|  | Procellariiformes | Diomedeidae | *Phoebastria* | *nigripes* |  |  |  | 18.8 |
| 202 | Procellariiformes | Diomedeidae | *Diomedea* | *epormophora* | 0.112 | 8595 | 9 | 42.3 |
|  | Procellariiformes | Diomedeidae | *Diomedea* | *exulans* |  |  |  | 23.3 |
| 203 | Procellariiformes | Diomedeidae | *Phoebetria* | *fusca* | 0.021 | 2705 | 18.84 | 30 |
|  | Procellariiformes | Diomedeidae | *Phoebetria* | *palpebrata* |  |  |  | 44 |
| 204 | Procellariiformes | Pelecanoididae | *Pelecanoides* | *magellani* | 0.073 | 127.5 | 9.7 | 11.7 |
|  | Procellariiformes | Pelecanoididae | *Pelecanoides* | *geogicus* |  |  |  | 11.4 |
| 205 | Piciformes | Picidae | *Picoides* | *mixtus* | 0.009 | 30.53 | 15.29 | 4.2 |
|  | Piciformes | Picidae | *Picoides* | *lignarius* |  |  |  | 4.2 |
| 206 | Piciformes | Picidae | *Picoides* | *scalaris* | 0.008 | 35.03 | 18.98 | 4.8 |
|  | Piciformes | Picidae | *Picoides* | *nuttallii* |  |  |  | 4.4 |
| 207 | Piciformes | Picidae | *Picus* | *viridanus* | 0.077 | 110.37 | 3.5 | 5.6 |
|  | Piciformes | Picidae | *Picus* | *vittatus* |  |  |  | 5.6 |
| 208 | Piciformes | Picidae | *Picus* | *mentalis* | 0.077 | 152.03 | 42.68 | 5.6 |
|  | Piciformes | Picidae | *Picus* | *flavinucha* |  |  |  | 5.6 |
| 209 | Piciformes | Picidae | *Picus* | *puniceus* | 0.049 | 70.2 | 16.88 | 5.6 |
|  | Piciformes | Picidae | *Picus* | *chlorolophus* |  |  |  | 5.6 |
| 210 | Piciformes | Picidae | *Picus* | *erythropygius* | 0.061 | 127.25 | 14.23 | 5.6 |
|  | Piciformes | Picidae | *Picus* | *canus* |  |  |  | 5.6 |
| 211 | Piciformes | Picidae | *Colaptes* | *auratus* | 0.09 | 132.5 | 2.47 | 4.3 |
|  | Piciformes | Picidae | *Colaptes* | *fernandinae* |  |  |  | 4.3 |
| 212 | Piciformes | Picidae | *Veniliornis* | *callonotus* | 0.034 | 30.23 | 29.39 | 4.2 |
|  | Piciformes | Picidae | *Veniliornis* | *dignus* |  |  |  | 4.2 |
| 213 | Piciformes | Picidae | *Veniliornis* | *passerinus* | 0.003 | 34.4 | 12.53 | 4.2 |
|  | Piciformes | Picidae | *Veniliornis* | *frontalis* |  |  |  | 4.2 |
| 214 | Piciformes | Picidae | *Veniliornis* | *chocoensis* | 0.083 | 29.4 | 4 | 4.2 |
|  | Piciformes | Picidae | *Veniliornis* | *kirkii* |  |  |  | 4.2 |
| 215 | Piciformes | Picidae | *Piculus* | *rubiginosus* | 0.033 | 59 | 14.68 | 4.3 |
|  | Piciformes | Picidae | *Piculus* | *auricularis* |  |  |  | 4.3 |
| 216 | Piciformes | Picidae | *Meiglyptes* | *tristis* | 0.07 | 42.4 | 40.3 | 3.5 |
|  | Piciformes | Picidae | *Meiglyptes* | *tukki* |  |  |  | 3.5 |
| 217 | Piciformes | Picidae | *Campephilus* | *principalis* | 0.02 | 516 |  | 6.5 |
|  | Piciformes | Picidae | *Campephilus* | *imperialis* |  |  |  | 6.5 |
| 218 | Piciformes | Picidae | *Campephilus* | *melanoleucos* | 0.056 | 249 | 5.47 | 6.5 |
|  | Piciformes | Picidae | *Campephilus* | *guatemalensis* |  |  |  | 6.5 |
| 219 | Piciformes | Ramphastidae | *Aulacorhynchus* | *derbianus* | 0.089 | 203.4 | 5.24 | 7 |
|  | Piciformes | Ramphastidae | *Aulacorhynchus* | *haematopygus* |  |  |  | 7 |
| 220 | Piciformes | Ramphastidae | *Rhamphastos* | *swainsonii* | 0.018 | 659 | 5.75 | 11.5 |
|  | Piciformes | Ramphastidae | *Rhamphastos* | *ambiguus* |  |  |  | 11.5 |
| 221 | Piciformes | Ramphastidae | *Rhamphastos* | *sulfuratus* | 0.098 | 479.06 | 33.72 | 11.5 |
|  | Piciformes | Ramphastidae | *Rhamphastos* | *toco* |  |  |  | 11.5 |
| 222 | Piciformes | Ramphastidae | *Capito* | *niger* | 0.046 | 56.53 | 14.97 | 8.5 |
|  | Piciformes | Ramphastidae | *Capito* | *auratus* |  |  |  | 8.5 |
| 223 | Piciformes | Ramphastidae | *Capito* | *maculicoronatus* | 0.024 | 56.13 | 10.71 | 8.5 |
|  | Piciformes | Ramphastidae | *Capito* | *squamatus* |  |  |  | 8.5 |
| 224 | Piciformes | Ramphastidae | *Semnornis* | *frantzii* | 0.1 | 77.5 | 41.35 | 8.5 |
|  | Piciformes | Ramphastidae | *Semnornis* | *ramphastinus* |  |  |  | 8.5 |
| 225 | Piciformes | Ramphastidae | *Andigena* | *cucullata* | 0.04 | 313 | 0.64 | 11.5 |
|  | Piciformes | Ramphastidae | *Andigena* | *hypoglauca* |  |  |  | 11.5 |
| 226 | Piciformes | Ramphastidae | *Stactolaema* | *leucotis* | 0.059 | 55.25 | 1.26 | 8.5 |
|  | Piciformes | Ramphastidae | *Stactolaema* | *whytii* |  |  |  | 8.5 |
| 227 | Piciformes | Ramphastidae | *Trachyphonus* | *darnaudii* | 0.115 | 47.65 | 49.94 | 8.5 |
|  | Piciformes | Ramphastidae | *Trachyphonus* | *erythrocephalus* |  |  |  | 8.5 |
| 228 | Psittaciformes | Psittacidae | *Pyrrhura* | *orcesi* | 0.031 | 72.9 | 5.6 | 6 |
|  | Psittaciformes | Psittacidae | *Pyrrhura* | *rupicola* |  |  |  | 6 |
| 229 | Psittaciformes | Psittacidae | *Pyrrhura* | *lepida* | 0.017 | 75 |  | 6 |
|  | Psittaciformes | Psittacidae | *Pyrrhura* | *perlata* |  |  |  | 6 |
| 230 | Psittaciformes | Psittacidae | *Pyrrhura* | *rhodocephala* | 0.019 | 82.2 |  | 6 |
|  | Psittaciformes | Psittacidae | *Pyrrhura* | *hoffmanni* |  |  |  | 6 |
| 231 | Psittaciformes | Psittacidae | *Pyrrhura* | *melanura* | 0.009 | 93.12 | 31.38 | 6 |
|  | Psittaciformes | Psittacidae | *Pyrrhura* | *albipectus* |  |  |  | 6 |
| 232 | Psittaciformes | Psittacidae | *Brotogeris* | *versicolurus* | 0.011 | 68.27 | 13.97 | 5 |
|  | Psittaciformes | Psittacidae | *Brotogeris* | *chiriri* |  |  |  | 5 |
| 233 | Psittaciformes | Psittacidae | *Brotogeris* | *cyanoptera* | 0.018 | 55.25 | 2.68 | 5 |
|  | Psittaciformes | Psittacidae | *Brotogeris* | *chrysoptera* |  |  |  | 5 |
| 234 | Psittaciformes | Psittacidae | *Brotogeris* | *pyrrhoptera* | 0.047 | 63.63 | 1.17 | 5 |
|  | Psittaciformes | Psittacidae | *Brotogeris* | *jugularis* |  |  |  | 5 |
| 235 | Psittaciformes | Psittacidae | *Pionus* | *sordidus* | 0.03 | 279.5 | 9.22 | 6.7 |
|  | Psittaciformes | Psittacidae | *Pionus* | *maximiliani* |  |  |  | 6.7 |
| 236 | Psittaciformes | Psittacidae | *Pionus* | *senilis* | 0.027 | 211 | 0.94 | 6.7 |
|  | Psittaciformes | Psittacidae | *Pionus* | *chalcopterus* |  |  |  | 6.7 |
| 237 | Psittaciformes | Psittacidae | *Psittacula* | *columboides* | 0.024 | 85.5 |  | 7.5 |
|  | Psittaciformes | Psittacidae | *Psittacula* | *calthropae* |  |  |  | 7.5 |
| 238 | Psittaciformes | Psittacidae | *Psittacula* | *cyanocephala* | 0.047 | 66 |  | 7.6 |
|  | Psittaciformes | Psittacidae | *Psittacula* | *roseata* |  |  |  | 7.5 |
| 239 | Psittaciformes | Psittacidae | *Psittacula* | *krameri* | 0.009 | 132 | 28.53 | 7.6 |
|  | Psittaciformes | Psittacidae | *Psittacula* | *echo* |  |  |  | 7.5 |
| 240 | Psittaciformes | Psittacidae | *Psittacula* | *derbiana* | 0.018 | 199 | 41.43 | 7.1 |
|  | Psittaciformes | Psittacidae | *Psittacula* | *alexandri* |  |  |  | 7.5 |
| 241 | Psittaciformes | Psittacidae | *Ara* | *macao* | 0.037 | 1114.5 | 16.39 | 12.7 |
|  | Psittaciformes | Psittacidae | *Ara* | *chloropterus* |  |  |  | 12.7 |
| 242 | Psittaciformes | Psittacidae | *Ara* | *ararauna* | 0.099 | 954.5 | 30.31 | 12.7 |
|  | Psittaciformes | Psittacidae | *Ara* | *glaucogularis* |  |  |  | 12.7 |
| 243 | Psittaciformes | Psittacidae | *Anodorhynchus* | *hyacinthinua* | 0.045 | 1135.5 | 29.38 | 10.4 |
|  | Psittaciformes | Psittacidae | *Anodorhynchus* | *leari* |  |  |  | 10.4 |
| 244 | Psittaciformes | Psittacidae | *Coracopsis* | *vasa* | 0.08 | 359.25 | 63.14 | 13.8 |
|  | Psittaciformes | Psittacidae | *Coracopsis* | *nigra* |  |  |  | 13.8 |
| 245 | Psittaciformes | Psittacidae | *Chalcopsitta* | *atra* | 0.04 | 195 |  | 6.8 |
|  | Psittaciformes | Psittacidae | *Chalcopsitta* | *duivenbodei* |  |  |  | 6.8 |
| 246 | Psittaciformes | Psittacidae | *Pezoporus* | *wallicus* | 0.107 | 71.35 |  | 9.7 |
|  | Psittaciformes | Psittacidae | *Pezoporus* | *occidentalis* |  |  |  | 9.7 |
| 247 | Psittaciformes | Psittacidae | *Primolius* | *maracana* | 0.032 | 250.5 | 4.3 | 10.2 |
|  | Psittaciformes | Psittacidae | *Primolius* | *auricollis* |  |  |  | 10.2 |
| 248 | Psittaciformes | Psittacidae | *Psittaculirostris* | *desmarestii* | 0.044 | 117 |  | 4.8 |
|  | Psittaciformes | Psittacidae | *Psittaculirostris* | *edwardsii* |  |  |  | 4.8 |
| 249 | Psittaciformes | Psittacidae | *Nestor* | *notabilis* | 0.044 | 648.25 | 50.55 | 12 |
|  | Psittaciformes | Psittacidae | *Nestor* | *meridionalis* |  |  |  | 12 |
| 250 | Psittaciformes | Cacatuidae | *Cacatua* | *sulphurea* | 0.03 | 461.07 | 60.78 | 13 |
|  | Psittaciformes | Cacatuidae | *Cacatua* | *galerita* |  |  |  | 13.8 |
| 251 | Psittaciformes | Cacatuidae | *Cacatua* | *alba* | 0.05 | 702.5 | 31.74 | 13 |
|  | Psittaciformes | Cacatuidae | *Cacatua* | *moluccensis* |  |  |  | 13 |
| 252 | Psittaciformes | Cacatuidae | *Cacatua* | *ducorpsii* | 0.042 | 481.33 | 24.95 | 13 |
|  | Psittaciformes | Cacatuidae | *Cacatua* | *sanguinea* |  |  |  | 13 |
| 253 | Falconiformes | Accipitridae | *Buteo* | *polyosoma* | 0.036 | 837 | 11.06 | 9.7 |
|  | Falconiformes | Accipitridae | *Buteo* | *albicaudatus* |  |  |  | 10 |
| 254 | Falconiformes | Accipitridae | *Buteo* | *lineatus* | 0.026 | 607 |  | 8.3 |
|  | Falconiformes | Accipitridae | *Buteo* | *ridgwayi* |  |  |  | 9.7 |
| 255 | Falconiformes | Accipitridae | *Buteo* | *regalis* | 0.015 | 1212.75 | 34.94 | 9.7 |
|  | Falconiformes | Accipitridae | *Buteo* | *lagopus* |  |  |  | 10 |
| 256 | Falconiformes | Accipitridae | *Buteo* | *augur* | 0.001 | 1250 | 27.45 | 9.7 |
|  | Falconiformes | Accipitridae | *Buteo* | *rufofuscus* |  |  |  | 10 |
| 257 | Falconiformes | Accipitridae | *Hieraaetus* | *fasciatus* | 0.016 | 1737.25 | 26.28 | 18 |
|  | Falconiformes | Accipitridae | *Hieraaetus* | *spilogaster* |  |  |  | 18 |
| 258 | Falconiformes | Accipitridae | *Hieraaetus* | *pennatus* | 0.028 | 812.75 | 5.21 | 18 |
|  | Falconiformes | Accipitridae | *Hieraaetus* | *morphnoides* |  |  |  | 18 |
| 259 | Falconiformes | Accipitridae | *Leucopternis* | *albicollis* | 0.021 | 693 | 6.98 | 7.6 |
|  | Falconiformes | Accipitridae | *Leucopternis* | *occidentalis* |  |  |  | 7.6 |
| 260 | Falconiformes | Accipitridae | *Leucopternis* | *melanops* | 0.021 | 340.13 | 7.57 | 7.6 |
|  | Falconiformes | Accipitridae | *Leucopternis* | *kuhli* |  |  |  | 7.6 |
| 261 | Falconiformes | Accipitridae | *Aquila* | *audax* | 0.028 | 3330.67 | 11.71 | 18.1 |
|  | Falconiformes | Accipitridae | *Aquila* | *gurneyi* |  |  |  | 16.8 |
| 262 | Falconiformes | Accipitridae | *Aquila* | *adalberti* | 0.024 | 2625 | 25 | 16.6 |
|  | Falconiformes | Accipitridae | *Aquila* | *rapax* |  |  |  | 16.6 |
| 263 | Falconiformes | Accipitridae | *Aquila* | *clanga* | 0.023 | 1787.75 | 37.88 | 16.6 |
|  | Falconiformes | Accipitridae | *Aquila* | *pomarina* |  |  |  | 16.6 |
| 264 | Falconiformes | Accipitridae | *Milvus* | *milvus* | 0.016 | 879.8 | 30.9 | 11.5 |
|  | Falconiformes | Accipitridae | *Milvus* | *migrans* |  |  |  | 11.5 |
| 265 | Falconiformes | Accipitridae | *Haliaeetus* | *albicilla* | 0.053 | 4766.5 | 1.11 | 17.5 |
|  | Falconiformes | Accipitridae | *Haliaeetus* | *leucocephalus* |  |  |  | 17.2 |
| 266 | Falconiformes | Accipitridae | *Haliaeetus* | *vocifer* | 0.027 | 2870.83 | 6.46 | 16.7 |
|  | Falconiformes | Accipitridae | *Haliaeetus* | *vociferoides* |  |  |  | 17.1 |
| 267 | Falconiformes | Accipitridae | *Haliaeetus* | *leucogaster* | 0.002 | 2527.5 | 23.56 | 14.7 |
|  | Falconiformes | Accipitridae | *Haliaeetus* | *sanfordi* |  |  |  | 17.1 |
| 268 | Falconiformes | Accipitridae | *Buteogallus* | *aequinoctialis* | 0.012 | 827.75 | 33.79 | 7.6 |
|  | Falconiformes | Accipitridae | *Buteogallus* | *anthracinus* |  |  |  | 7.6 |
| 269 | Falconiformes | Accipitridae | *Chondrohierax* | *uncinatus* | 0.018 | 287 |  | 7.6 |
|  | Falconiformes | Accipitridae | *Chondrohierax* | *wilsoni* |  |  |  | 7.6 |
| 270 | Falconiformes | Accipitridae | *Circaetus* | *fasciolatus* | 0.038 | 1017 |  | 12.9 |
|  | Falconiformes | Accipitridae | *Circaetus* | *cinerascens* |  |  |  | 12.9 |
| 271 | Falconiformes | Accipitridae | *Spilornis* | *rufipectus* | 0.025 | 705 |  | 12.9 |
|  | Falconiformes | Accipitridae | *Spilornis* | *holospilus* |  |  |  | 12.9 |
| 272 | Falconiformes | Accipitridae | *Ichthyophaga* | *humilis* | 0.075 | 1619 | 61.62 | 17.3 |
|  | Falconiformes | Accipitridae | *Ichthyophaga* | *ichthyaetus* |  |  |  | 17.3 |
| 273 | Falconiformes | Accipitridae | *Melierax* | *poliopterus* | 0.038 | 649 | 5.78 | 8.7 |
|  | Falconiformes | Accipitridae | *Melierax* | *canorus* |  |  |  | 8.7 |
| 274 | Falconiformes | Accipitridae | *Butastur* | *teesa* | 0.057 | 385 | 21.69 | 6.6 |
|  | Falconiformes | Accipitridae | *Butastur* | *indicus* |  |  |  | 6.6 |
| 275 | Falconiformes | Accipitridae | *Haliastur* | *sphenurus* | 0.05 | 641 | 29.75 | 11.5 |
|  | Falconiformes | Accipitridae | *Haliastur* | *indus* |  |  |  | 11.5 |
| 276 | Falconiformes | Accipitridae | *Harpyhaliaetus* | *solitarius* | 0.013 | 2975 | 1.67 | 10.5 |
|  | Falconiformes | Accipitridae | *Harpyhaliaetus* | *coronatus* |  |  |  | 10.5 |
| 277 | Falconiformes | Accipitridae | *Ictinia* | *mississippiensis* | 0.042 | 278 | 21.22 | 6.6 |
|  | Falconiformes | Accipitridae | *Ictinia* | *plumbea* |  |  |  | 6.6 |
| 278 | Falconiformes | Falconidae | *Falco* | *peregrinus* | 0.002 | 714.96 | 20.52 | 6.8 |
|  | Falconiformes | Falconidae | *Falco* | *pelegrinoides* |  |  |  | 6.4 |
| 279 | Falconiformes | Falconidae | *Falco* | *rusticolus* | 0.006 | 1038.5 | 57.84 | 6.4 |
|  | Falconiformes | Falconidae | *Falco* | *biarmicus* |  |  |  | 6.4 |
| 280 | Falconiformes | Falconidae | *Falco* | *vespertinus* | 0.012 | 147.25 | 6.89 | 5.7 |
|  | Falconiformes | Falconidae | *Falco* | *amurensis* |  |  |  | 6.2 |
| 281 | Falconiformes | Falconidae | *Falco* | *subbuteo* | 0.01 | 196.25 | 12.65 | 6.4 |
|  | Falconiformes | Falconidae | *Falco* | *cuvierii* |  |  |  | 6.4 |
| 282 | Falconiformes | Falconidae | *Falco* | *sparverius* | 0.071 | 229.25 | 66.52 | 5.7 |
|  | Falconiformes | Falconidae | *Falco* | *femoralis* |  |  |  | 6.2 |
| 283 | Falconiformes | Falconidae | *Falco* | *tinnunculus* | 0.012 | 178.1 | 6.41 | 5.4 |
|  | Falconiformes | Falconidae | *Falco* | *cenchroides* |  |  |  | 6.2 |
| 284 | Falconiformes | Falconidae | *Falco* | *araeus* | 0.009 | 102.58 | 35.88 | 6.2 |
|  | Falconiformes | Falconidae | *Falco* | *newtoni* |  |  |  | 6.2 |
| 285 | Falconiformes | Falconidae | *Milvago* | *chimachima* | 0 | 309 | 6.18 | 10.3 |
|  | Falconiformes | Falconidae | *Milvago* | *chimango* |  |  |  | 10.3 |
| 286 | Charadriiformes | Laridae | *Chlidonias* | *leucopterus* | 0.025 | 59.75 | 17 | 9.9 |
|  | Charadriiformes | Laridae | *Chlidonias* | *niger* |  |  |  | 8.8 |
| 287 | Charadriiformes | Laridae | *Anous* | *tenuirostris* | 0.068 | 111.15 | 1.52 | 11.5 |
|  | Charadriiformes | Laridae | *Anous* | *minutus* |  |  |  | 10.8 |
| 288 | Charadriiformes | Scolopacidae | *Limosa* | *fedoa* | 0.072 | 307 | 28.73 | 8.7 |
|  | Charadriiformes | Scolopacidae | *Limosa* | *haemastica* |  |  |  | 8.7 |
| 289 | Charadriiformes | Scolopacidae | *Phalaropus* | *tricolor* | 0.168 | 48.35 | 38.7 | 5.7 |
|  | Charadriiformes | Scolopacidae | *Phalaropus* | *lobatus* |  |  |  | 4.9 |
| 290 | Charadriiformes | Stercorariidae | *Stercorarius* | *parasiticus* | 0.078 | 367 | 35.24 | 13.5 |
|  | Charadriiformes | Stercorariidae | *Stercorarius* | *longicaudus* |  |  |  | 12.5 |
| 291 | Charadriiformes | Stercorariidae | *Stercorarius* | *skua* | 0 | 1017 | 48.21 | 17.5 |
|  | Charadriiformes | Stercorariidae | *Stercorarius* | *pomarinus* |  |  |  | 13.5 |
| 292 | Charadriiformes | Stercorariidae | *Stercorarius* | *maccormicki* | 0.002 | 1558.65 | 23.71 | 17.1 |
|  | Charadriiformes | Stercorariidae | *Stercorarius* | *antarcticus* |  |  |  | 17.1 |
| 293 | Charadriiformes | Alcidae | *Uria* | *lomvia* | 0.069 | 985 | 2.82 | 14 |
|  | Charadriiformes | Alcidae | *Uria* | *aalge* |  |  |  | 15.1 |
| 294 | Charadriiformes | Alcidae | *Cepphus* | *columba* | 0.012 | 510 | 7.55 | 8 |
|  | Charadriiformes | Alcidae | *Cepphus* | *carbo* |  |  |  | 9.2 |
| 295 | Charadriiformes | Alcidae | *Synthliboramphus* | *hypoleucus* | 0.01 | 159 | 9.58 | 12.1 |
|  | Charadriiformes | Alcidae | *Synthliboramphus* | *craveri* |  |  |  | 12.1 |
| 296 | Charadriiformes | Alcidae | *Synthliboramphus* | *antiquus* | 0.044 | 202.5 | 4.35 | 7.3 |
|  | Charadriiformes | Alcidae | *Synthliboramphus* | *wumizusume* |  |  |  | 12.1 |
| 297 | Charadriiformes | Alcidae | *Aethia* | *pusilla* | 0.055 | 98 | 25 | 6.9 |
|  | Charadriiformes | Alcidae | *Aethia* | *pygmaea* |  |  |  | 7.6 |
| 298 | Charadriiformes | Jacanidae | *Jacana* | *spinosa* | 0.005 | 101.95 | 11.99 | 4.8 |
|  | Charadriiformes | Jacanidae | *Jacana* | *jacana* |  |  |  | 4.8 |
| 299 | Galliformes | Phasianidae | *Ptilopachus* | *petrosus* | 0.099 | 193 |  | 3.9 |
|  | Galliformes | Phasianidae | *Ptilopachus* | *nahani* |  |  |  | 3.9 |
| 300 | Galliformes | Phasianidae | *Tympanuchus* | *pallidicinctus* | 0.005 | 805.53 | 13.76 | 5.5 |
|  | Galliformes | Phasianidae | *Tympanuchus* | *cupido* |  |  |  | 5.5 |
| 301 | Galliformes | Phasianidae | *Lophophorus* | *impejanus* | 0.056 | 2387.67 | 30.93 | 5.2 |
|  | Galliformes | Phasianidae | *Lophophorus* | *lhuysii* |  |  |  | 5.2 |
| 302 | Galliformes | Phasianidae | *Tetraophasis* | *obscurus* | 0.027 | 964.5 | 19.72 | 5 |
|  | Galliformes | Phasianidae | *Tetraophasis* | *szechenyii* |  |  |  | 5 |
| 303 | Galliformes | Phasianidae | *Pavo* | *cristatus* | 0.033 | 3480.75 | 34.01 | 6.1 |
|  | Galliformes | Phasianidae | *Pavo* | *muticus* |  |  |  | 6.1 |
| 304 | Galliformes | Phasianidae | *Bambusicola* | *fytchii* | 0.09 | 294.38 | 15.15 | 2.8 |
|  | Galliformes | Phasianidae | *Bambusicola* | *throacicus* |  |  |  | 2.8 |
| 305 | Galliformes | Phasianidae | *Crossoptilon* | *crossoptilon* | 0.009 | 2137.5 |  | 5 |
|  | Galliformes | Phasianidae | *Crossoptilon* | *harmani* |  |  |  | 5 |
| 306 | Galliformes | Phasianidae | *Crossoptilon* | *mantchuricum* | 0.001 | 1842.5 | 6.05 | 5 |
|  | Galliformes | Phasianidae | *Crossoptilon* | *auritum* |  |  |  | 5 |
| 307 | Galliformes | Phasianidae | *Perdix* | *dauurica* | 0.048 | 299.67 | 29.17 | 3.9 |
|  | Galliformes | Phasianidae | *Perdix* | *hodgsoniae* |  |  |  | 3.9 |
| 308 | Galliformes | Phasianidae | *Tragopan* | *temminckii* | 0.038 | 1167 | 2.87 | 5.2 |
|  | Galliformes | Phasianidae | *Tragopan* | *caboti* |  |  |  | 5.2 |
| 309 | Galliformes | Phasianidae | *Polyplectron* | *malacense* | 0.043 | 608 |  | 5 |
|  | Galliformes | Phasianidae | *Polyplectron* | *schleiermacheri* |  |  |  | 5 |
| 310 | Galliformes | Phasianidae | *Polyplectron* | *bicalcaratum* | 0.014 | 453 | 27.51 | 5 |
|  | Galliformes | Phasianidae | *Polyplectron* | *chalcurum* |  |  |  | 5 |
| 311 | Galliformes | Phasianidae | *Chrysolophus* | *pictus* | 0.03 | 656.63 | 22.11 | 5 |
|  | Galliformes | Phasianidae | *Chrysolophus* | *amherstiae* |  |  |  | 5 |
| 312 | Galliformes | Phasianidae | *Syrmaticus* | *ellioti* | 0.01 | 952.88 | 12.61 | 5 |
|  | Galliformes | Phasianidae | *Syrmaticus* | *humiae* |  |  |  | 5 |
| 313 | Galliformes | Phasianidae | *Lophura* | *ignita* | 0.044 | 1471.25 | 37.88 | 5 |
|  | Galliformes | Phasianidae | *Lophura* | *diardi* |  |  |  | 5 |
| 314 | Galliformes | Phasianidae | *Lophura* | *leucomelanos* | 0.027 | 1192.25 | 39.97 | 5 |
|  | Galliformes | Phasianidae | *Lophura* | *nycthemera* |  |  |  | 5 |
| 315 | Galliformes | Phasianidae | *Lophura* | *edwardsi* | 0.001 | 1091 | 1.64 | 5 |
|  | Galliformes | Phasianidae | *Lophura* | *hatinhensis* |  |  |  | 5 |
| 316 | Galliformes | Phasianidae | *Tetrao* | *urogallus* | 0.05 | 2953.75 | 0.25 | 6.4 |
|  | Galliformes | Phasianidae | *Tetrao* | *parvirostris* |  |  |  | 6.4 |
| 317 | Galliformes | Phasianidae | *Lyrurus* | *tetrix* | 0.034 | 949 | 24.67 | 6.4 |
|  | Galliformes | Phasianidae | *Lyrurus* | *mlokosiewiczi* |  |  |  | 6.4 |
| 318 | Galliformes | Phasianidae | *Tetraogallus* | *himalayensis* | 0.02 | 2656 | 12.35 | 5 |
|  | Galliformes | Phasianidae | *Tetraogallus* | *altaicus* |  |  |  | 5 |
| 319 | Galliformes | Phasianidae | *Gallus* | *gallus* | 0.021 | 811.5 | 10.23 | 5 |
|  | Galliformes | Phasianidae | *Gallus* | *sonneratii* |  |  |  | 5 |
| 320 | Galliformes | Phasianidae | *Alectoris* | *magna* | 0.04 | 515.67 | 20.25 | 3.9 |
|  | Galliformes | Phasianidae | *Alectoris* | *philbyi* |  |  |  | 3.9 |
| 321 | Galliformes | Phasianidae | *Alectoris* | *melanocephala* | 0.069 | 520.75 | 32.83 | 3.9 |
|  | Galliformes | Phasianidae | *Alectoris* | *barbara* |  |  |  | 3.9 |
| 322 | Galliformes | Cracidae | *Crax* | *fasciolata* | 0.019 | 2924 | 15.75 | 11.5 |
|  | Galliformes | Cracidae | *Crax* | *alector* |  |  |  | 11.5 |
| 323 | Galliformes | Cracidae | *Mitu* | *salvini* | 0.025 | 2908.67 | 9.26 | 10.5 |
|  | Galliformes | Cracidae | *Mitu* | *tuberosum* |  |  |  | 11.5 |
| 324 | Galliformes | Cracidae | *Pauxi* | *pauxi* | 0.036 | 3447.5 | 15.89 | 13.5 |
|  | Galliformes | Cracidae | *Pauxi* | *unicornis* |  |  |  | 14.5 |
| 325 | Galliformes | Odontophoridae | *Callipepla* | *californica* | 0.024 | 166.2 | 0.2 | 4 |
|  | Galliformes | Odontophoridae | *Callipepla* | *gambelii* |  |  |  | 4 |
| 326 | Galliformes | Numididae | *Guttera* | *plumifera* | 0.05 | 1113 | 28.98 | 3.9 |
|  | Galliformes | Numididae | *Guttera* | *pucherani* |  |  |  | 3.9 |
| 327 | Passeriformes | Stenostiridae | *Culicicapa* | *ceylonensis* | 0.064 | 8.05 | 8.33 | 3.2 |
|  | Passeriformes | Stenostiridae | *Culicicapa* | *helianthea* |  |  |  | 3.2 |
| 328 | Passeriformes | Orthonychidae | *Orthonyx* | *temminckii* | 0.142 | 108.07 | 63.19 | 4.4 |
|  | Passeriformes | Orthonychidae | *Orthonyx* | *spaldingii* |  |  |  | 4.4 |
| 329 | Passeriformes | Bombycillidae | *Ptilogonys* | *cinereus* | 0.07 | 35.4 | 7.44 | 5.6 |
|  | Passeriformes | Bombycillidae | *Ptilogonys* | *caudatus* |  |  |  | 5.6 |
| 330 | Passeriformes | Maluridae | *Stipiturus* | *mallee* | 0.028 | 5.25 | 9.09 | 9.7 |
|  | Passeriformes | Maluridae | *Stipiturus* | *ruficeps* |  |  |  | 9.7 |
| 331 | Passeriformes | Ptilonorhynchidae | *Chlamydera* | *nuchalis* | 0.031 | 178.75 | 22.14 | 6.7 |
|  | Passeriformes | Ptilonorhynchidae | *Chlamydera* | *cerviniventris* |  |  |  | 6.7 |
| 332 | Passeriformes | Ptilonorhynchidae | *Sericulus* | *aureus* | 0.044 | 172 |  | 10.6 |
|  | Passeriformes | Ptilonorhynchidae | *Sericulus* | *ardens* |  |  |  | 10.6 |
| 333 | Passeriformes | Petroicidae | *Amalocichla* | *sclateriana* | 0.127 | 32.7 |  | 5.2 |
|  | Passeriformes | Petroicidae | *Amalocichla* | *incerta* |  |  |  | 5.2 |
| 334 | Passeriformes | Petroicidae | *Petroica* | *macrocephala* | 0.04 | 15.7 | 49.79 | 6.5 |
|  | Passeriformes | Petroicidae | *Petroica* | *traversi* |  |  |  | 7 |
| 335 | Passeriformes | Colluricinclidae | *Pitohui* | *incertus* | 0.079 | 94 |  | 7.9 |
|  | Passeriformes | Colluricinclidae | *Pitohui* | *ferrugineus* |  |  |  | 7.9 |
| 336 | Passeriformes | Colluricinclidae | *Pitohui* | *kirhocephalus* | 0.071 | 82.45 | 22.5 | 7.9 |
|  | Passeriformes | Colluricinclidae | *Pitohui* | *dichrous* |  |  |  | 7.9 |
| 337 | Passeriformes | Monarchidae | *Trochocercus* | *cyanomelas* | 0.049 | 11 | 13.56 | 4.2 |
|  | Passeriformes | Monarchidae | *Trochocercus* | *nitens* |  |  |  | 4.2 |
| 338 | Passeriformes | Monarchidae | *Pomarea* | *dimidiata* | 0.052 | 24.2 | 9.45 | 6.9 |
|  | Passeriformes | Monarchidae | *Pomarea* | *nigra* |  |  |  | 6.9 |
| 339 | Passeriformes | Monarchidae | *Pomarea* | *mendozae* | 0.042 | 26.75 | 19.26 | 6.9 |
|  | Passeriformes | Monarchidae | *Pomarea* | *iphis* |  |  |  | 6.9 |
| 340 | Passeriformes | Estrildidae | *Poephila* | *acuticauda* | 0.033 | 13.45 | 19.46 | 3 |
|  | Passeriformes | Estrildidae | *Poephila* | *cincta* |  |  |  | 3 |
| 341 | Passeriformes | Estrildidae | *Taeniopygia* | *guttata* | 0.069 | 11.23 | 20.33 | 3 |
|  | Passeriformes | Estrildidae | *Taeniopygia* | *bichenovii* |  |  |  | 3 |
| 342 | Passeriformes | Paradisaeidae | *Paradigalla* | *carunculata* | 0.056 | 168.67 | 1.18 | 7.9 |
|  | Passeriformes | Paradisaeidae | *Paradigalla* | *brevicauda* |  |  |  | 7.9 |
| 343 | Passeriformes | Paradisaeidae | *Astrapia* | *nigra* | 0.038 | 151 | 30.79 | 7.9 |
|  | Passeriformes | Paradisaeidae | *Astrapia* | *splendidissima* |  |  |  | 7.9 |
| 344 | Passeriformes | Paradisaeidae | *Astrapia* | *mayeri* | 0.002 | 140.75 | 1.76 | 7.9 |
|  | Passeriformes | Paradisaeidae | *Astrapia* | *stephaniae* |  |  |  | 7.9 |
| 345 | Passeriformes | Paradisaeidae | *Epimachus* | *fastuosus* | 0.066 | 231.31 | 3.66 | 7.9 |
|  | Passeriformes | Paradisaeidae | *Epimachus* | *mayeri* |  |  |  | 7.9 |
| 346 | Passeriformes | Paradisaeidae | *Ptiloris* | *paradiseus* | 0.026 | 108 | 20.75 | 6 |
|  | Passeriformes | Paradisaeidae | *Ptiloris* | *victoriae* |  |  |  | 6 |
| 347 | Passeriformes | Paradisaeidae | *Parotia* | *lawesii* | 0.006 | 161.75 | 7.44 | 7.9 |
|  | Passeriformes | Paradisaeidae | *Parotia* | *helenae* |  |  |  | 7.9 |
| 348 | Passeriformes | Paradisaeidae | *Parotia* | *sefilata* | 0.101 | 176.81 | 5.7 | 7.9 |
|  | Passeriformes | Paradisaeidae | *Parotia* | *carolae* |  |  |  | 7.9 |
| 349 | Passeriformes | Paradisaeidae | *Manucodia* | *chalybatus* | 0.065 | 324.5 | 50.12 | 7.9 |
|  | Passeriformes | Paradisaeidae | *Manucodia* | *comrii* |  |  |  | 7.9 |
| 350 | Passeriformes | Paradisaeidae | *Paradisaea* | *apoda* | 0.008 | 204.2 | 21.93 | 9 |
|  | Passeriformes | Paradisaeidae | *Paradisaea* | *raggiana* |  |  |  | 9 |
| 351 | Passeriformes | Paradisaeidae | *Paradisaea* | *decora* | 0.031 | 198.67 | 24.26 | 9 |
|  | Passeriformes | Paradisaeidae | *Paradisaea* | *rubra* |  |  |  | 9 |
| 352 | Passeriformes | Formicariidae | *Pittasoma* | *michleri* | 0.135 | 102.75 | 11.47 | 3.4 |
|  | Passeriformes | Formicariidae | *Pittasoma* | *rufopileatum* |  |  |  | 3.4 |
| 353 | Passeriformes | Formicariidae | *Myrmothera* | *campanisona* | 0.059 | 49.7 | 10.31 | 2.9 |
|  | Passeriformes | Formicariidae | *Myrmothera* | *simplex* |  |  |  | 2.9 |
| 354 | Passeriformes | Meliphagidae | *Timeliopsis* | *fulvigula* | 0.131 | 25.15 | 42.81 | 5.8 |
|  | Passeriformes | Meliphagidae | *Timeliopsis* | *griseigula* |  |  |  | 5.8 |
| 355 | Passeriformes | Meliphagidae | *Ramsayornis* | *modestus* | 0.087 | 12.53 | 5.1 | 5.8 |
|  | Passeriformes | Meliphagidae | *Ramsayornis* | *fasciatus* |  |  |  | 5.8 |
| 356 | Passeriformes | Meliphagidae | *Conopophila* | *albogularis* | 0.084 | 11.3 | 8.47 | 5.1 |
|  | Passeriformes | Meliphagidae | *Conopophila* | *rufogularis* |  |  |  | 5.1 |
| 357 | Passeriformes | Meliphagidae | *Epthianura* | *tricolor* | 0.046 | 10.6 | 1.87 | 5.8 |
|  | Passeriformes | Meliphagidae | *Epthianura* | *aurifrons* |  |  |  | 5.8 |
| 358 | Passeriformes | Meliphagidae | *Anthochaera* | *carunculata* | 0.121 | 125.93 | 26.91 | 6.9 |
|  | Passeriformes | Meliphagidae | *Anthochaera* | *paradoxa* |  |  |  | 6.9 |
| 359 | Passeriformes | Meliphagidae | *Phylidonyris* | *nigra* | 0.04 | 19.25 | 9.88 | 6.2 |
|  | Passeriformes | Meliphagidae | *Phylidonyris* | *novaehollandiae* |  |  |  | 6.2 |
| 360 | Passeriformes | Meliphagidae | *Manorina* | *melanocephala* | 0.008 | 57.82 | 8.24 | 7.1 |
|  | Passeriformes | Meliphagidae | *Manorina* | *flavigula* |  |  |  | 7.1 |
| 361 | Passeriformes | Aegithalidae | *Aegithalos* | *leucogenys* | 0.083 | 6.53 | 9.63 | 4.2 |
|  | Passeriformes | Aegithalidae | *Aegithalos* | *concinnus* |  |  |  | 4.2 |
| 362 | Passeriformes | Aegithalidae | *Aegithalos* | *bonvaloti* | 0.003 | 6 |  | 4.2 |
|  | Passeriformes | Aegithalidae | *Aegithalos* | *fuliginosus* |  |  |  | 4.2 |
| 363 | Passeriformes | Dicruridae | *Dicrurus* | *bracteatus* | 0.109 | 101.57 | 32.8 | 2.8 |
|  | Passeriformes | Dicruridae | *Dicrurus* | *magarhynchus* |  |  |  | 2.8 |
| 364 | Passeriformes | Dicruridae | *Dicrurus* | *balicassius* | 0.094 | 74.6 | 8.71 | 2.8 |
|  | Passeriformes | Dicruridae | *Dicrurus* | *hottentottus* |  |  |  | 2.8 |
| 365 | Passeriformes | Dicruridae | *Dicrurus* | *aeneus* | 0.122 | 32.03 | 38.52 | 2.8 |
|  | Passeriformes | Dicruridae | *Dicrurus* | *remifer* |  |  |  | 2.8 |
| 366 | Passeriformes | Dicruridae | *Dicrurus* | *ludwigii* | 0.112 | 34.15 | 24.42 | 2.8 |
|  | Passeriformes | Dicruridae | *Dicrurus* | *atripennis* |  |  |  | 2.8 |
| 367 | Passeriformes | Dicruridae | *Dicrurus* | *aldabranus* | 0.007 | 48.03 | 2.89 | 2.8 |
|  | Passeriformes | Dicruridae | *Dicrurus* | *forficatus* |  |  |  | 2.8 |
| 368 | Passeriformes | Cotingidae | *Snowornis* | *subalaris* | 0.143 | 77.4 | 9.36 | 4.6 |
|  | Passeriformes | Cotingidae | *Snowornis* | *cryptolophus* |  |  |  | 4.6 |
| 369 | Passeriformes | Cotingidae | *Rupicola* | *rupicola* | 0.091 | 219.5 | 20.08 | 4.6 |
|  | Passeriformes | Cotingidae | *Rupicola* | *peruvianus* |  |  |  | 4.6 |
| 370 | Passeriformes | Motacillidae | *Motacilla* | *clara* | 0.03 | 22.45 | 33.7 | 6.5 |
|  | Passeriformes | Motacillidae | *Motacilla* | *aguimp* |  |  |  | 4.7 |
| 371 | Passeriformes | Alaudidae | *Ammomanes* | *cinctura* | 0.105 | 25.6 |  | 3.8 |
|  | Passeriformes | Alaudidae | *Ammomanes* | *phoenicura* |  |  |  | 3.8 |
| 372 | Passeriformes | Alaudidae | *Eremophila* | *alpestris* | 0.043 | 35.39 | 10.76 | 4.5 |
|  | Passeriformes | Alaudidae | *Eremophila* | *bilopha* |  |  |  | 4.5 |
| 373 | Passeriformes | Alaudidae | *Certhilauda* | *erythrochlamys* | 0.021 | 28.93 | 5.55 | 3.5 |
|  | Passeriformes | Alaudidae | *Certhilauda* | *barlowi* |  |  |  | 3.5 |
| 374 | Passeriformes | Alaudidae | *Certhilauda* | *curvirostris* | 0.024 | 41.9 |  | 3.5 |
|  | Passeriformes | Alaudidae | *Certhilauda* | *brevirostris* |  |  |  | 3.5 |
| 375 | Passeriformes | Cinclidae | *Cinclus* | *pallasii* | 0.029 | 63.41 | 24.86 | 4 |
|  | Passeriformes | Cinclidae | *Cinclus* | *mexicanus* |  |  |  | 3.9 |
| 376 | Passeriformes | Hirundinidae | *Phedina* | *borborica* | 0.167 | 13 |  | 4.1 |
|  | Passeriformes | Hirundinidae | *Phedina* | *brazzae* |  |  |  | 4.1 |
| 377 | Passeriformes | Hirundinidae | *Atticora* | *fasciata* | 0.095 | 11.9 | 16.92 | 4.1 |
|  | Passeriformes | Hirundinidae | *Atticora* | *melanoleuca* |  |  |  | 4.1 |
| 378 | Passeriformes | Hirundinidae | *Notiochelidon* | *murina* | 0.074 | 10.75 | 16.24 | 4.1 |
|  | Passeriformes | Hirundinidae | *Notiochelidon* | *flavipes* |  |  |  | 4.1 |
| 379 | Passeriformes | Hirundinidae | *Cecropsis* | *abyssinica* | 0.082 | 30.1 |  | 4 |
|  | Passeriformes | Hirundinidae | *Cecropsis* | *semirufa* |  |  |  | 4 |
| 380 | Passeriformes | Hirundinidae | *Tachycineta* | *leucorrhoa* | 0.033 | 15.1 | 2.61 | 4.1 |
|  | Passeriformes | Hirundinidae | *Tachycineta* | *meyeni* |  |  |  | 4.1 |
| 381 | Passeriformes | Hirundinidae | *Tachycineta* | *albilinea* | 0.051 | 17.2 | 5.65 | 4.1 |
|  | Passeriformes | Hirundinidae | *Tachycineta* | *albiventer* |  |  |  | 4.1 |
| 382 | Passeriformes | Hirundinidae | *Petrochelidon* | *fulva* | 0.017 | 24.13 |  | 4.5 |
|  | Passeriformes | Hirundinidae | *Petrochelidon* | *rufocollaris* |  |  |  | 4.4 |
| 383 | Passeriformes | Hirundinidae | *Hirundo* | *atrocaerulea* | 0.058 | 14 | 2.82 | 4 |
|  | Passeriformes | Hirundinidae | *Hirundo* | *nigrorufa* |  |  |  | 4 |
| 384 | Passeriformes | Hirundinidae | *Hirundo* | *dimidiata* | 0.011 | 11.55 | 9.09 | 4 |
|  | Passeriformes | Hirundinidae | *Hirundo* | *megaensis* |  |  |  | 4 |
| 385 | Passeriformes | Pycnonotidae | *Xanthomixis* | *apperti* | 0.064 | 16.4 | 19.78 | 3.6 |
|  | Passeriformes | Pycnonotidae | *Xanthomixis* | *cinereiceps* |  |  |  | 3.6 |
| 386 | Passeriformes | Troglodytidae | *Henicorhina* | *leucosticta* | 0.088 | 15.66 | 14.66 | 3.6 |
|  | Passeriformes | Troglodytidae | *Henicorhina* | *leucophrys* |  |  |  | 3.6 |
| 387 | Passeriformes | Troglodytidae | *Campylorhynchus* | *albobrunneus* | 0.056 | 31.45 | 8.81 | 3.6 |
|  | Passeriformes | Troglodytidae | *Campylorhynchus* | *fasciatus* |  |  |  | 3.6 |
| 388 | Passeriformes | Troglodytidae | *Campylorhynchus* | *rufinucha* | 0.059 | 30.55 | 2.9 | 3.6 |
|  | Passeriformes | Troglodytidae | *Campylorhynchus* | *gularis* |  |  |  | 3.6 |
| 389 | Passeriformes | Troglodytidae | *Campylorhynchus* | *chiapensis* | 0.054 | 46.65 | 16.7 | 3.6 |
|  | Passeriformes | Troglodytidae | *Campylorhynchus* | *griseus* |  |  |  | 3.6 |
| 390 | Passeriformes | Estrildidae | *Neochmia* | *ruficauda* | 0.067 | 11.95 | 12.55 | 3 |
|  | Passeriformes | Estrildidae | *Neochmia* | *modesta* |  |  |  | 3 |
| 391 | Passeriformes | Estrildidae | *Amadina* | *erythrocephala* | 0.027 | 20.25 | 20 | 3 |
|  | Passeriformes | Estrildidae | *Amadina* | *fasciata* |  |  |  | 3 |
| 392 | Passeriformes | Ploceidae | *Euplectes* | *hordeaceus* | 0.03 | 17.57 | 28.35 | 3.8 |
|  | Passeriformes | Ploceidae | *Euplectes* | *diadematus* |  |  |  | 3.8 |
| 393 | Passeriformes | Ploceidae | *Euplectes* | *orix* | 0.028 | 19.57 | 45.89 | 4.1 |
|  | Passeriformes | Ploceidae | *Euplectes* | *nigroventris* |  |  |  | 3.8 |
| 394 | Passeriformes | Ploceidae | *Euplectes* | *albonotatus* | 0.036 | 23.2 | 16.24 | 3.8 |
|  | Passeriformes | Ploceidae | *Euplectes* | *axillaris* |  |  |  | 3.8 |
| 395 | Passeriformes | Ploceidae | *Euplectes* | *psammocromius* | 0.062 | 35.3 | 1.13 | 3.8 |
|  | Passeriformes | Ploceidae | *Euplectes* | *progne* |  |  |  | 3.8 |
| 396 | Passeriformes | Passeridae | *Montifringilla* | *nivalis* | 0.065 | 36.9 |  | 2.8 |
|  | Passeriformes | Passeridae | *Montifringilla* | *henrici* |  |  |  | 2.8 |
| 397 | Passeriformes | Tyrannidae | *Mitrephanes* | *phaeocercus* | 0.064 | 9.07 | 14 | 3.6 |
|  | Passeriformes | Tyrannidae | *Mitrephanes* | *olivaceus* |  |  |  | 3.6 |
| 398 | Passeriformes | Tyrannidae | *Corythopis* | *torquatus* | 0.109 | 14.65 | 4.67 | 3.6 |
|  | Passeriformes | Tyrannidae | *Corythopis* | *delalandi* |  |  |  | 3.6 |
| 399 | Passeriformes | Tyrannidae | *Anairetes* | *nigrocristatus* | 0.013 | 5.9 |  | 3.6 |
|  | Passeriformes | Tyrannidae | *Anairetes* | *reguloides* |  |  |  | 3.6 |
| 400 | Passeriformes | Tyrannidae | *Anairetes* | *alpinus* | 0.034 | 7.5 | 33.33 | 3.6 |
|  | Passeriformes | Tyrannidae | *Anairetes* | *flavirostris* |  |  |  | 3.6 |
| 401 | Passeriformes | Tyrannidae | *Anairetes* | *parulus* | 0.021 | 6.2 |  | 3.6 |
|  | Passeriformes | Tyrannidae | *Anairetes* | *fernandezianus* |  |  |  | 3.6 |
| 402 | Passeriformes | Tyrannidae | *Sayornis* | *nigricans* | 0.033 | 19 | 5.33 | 3.8 |
|  | Passeriformes | Tyrannidae | *Sayornis* | *phoebe* |  |  |  | 3.4 |
| 403 | Passeriformes | Tyrannidae | *Mionectes* | *oleagineus* | 0.045 | 12.6 | 13.33 | 4 |
|  | Passeriformes | Tyrannidae | *Mionectes* | *macconnelli* |  |  |  | 4 |
| 404 | Passeriformes | Tyrannidae | *Mionectes* | *striaticollis* | 0.072 | 15 | 0 | 4 |
|  | Passeriformes | Tyrannidae | *Mionectes* | *olivaceus* |  |  |  | 4 |
| 405 | Passeriformes | Tyrannidae | *Tityra* | *cayana* | 0.077 | 73.7 | 14.12 | 4.6 |
|  | Passeriformes | Tyrannidae | *Tityra* | *semifasciata* |  |  |  | 4.6 |
| 406 | Passeriformes | Dendrocolaptidae | *Dendrocolaptes* | *sanctithomae* | 0.064 | 67.8 | 2.62 | 3.8 |
|  | Passeriformes | Dendrocolaptidae | *Dendrocolaptes* | *certhia* |  |  |  | 3.8 |
| 407 | Passeriformes | Dendrocolaptidae | *Campylorhamphus* | *procurvoides* | 0.042 | 37.15 | 17.89 | 4 |
|  | Passeriformes | Dendrocolaptidae | *Campylorhamphus* | *trochilirostris* |  |  |  | 4 |
| 408 | Passeriformes | Dendrocolaptidae | *Dendrocincla* | *fuliginosa* | 0.05 | 35.8 | 11.24 | 5.5 |
|  | Passeriformes | Dendrocolaptidae | *Dendrocincla* | *anabatina* |  |  |  | 5.4 |
| 409 | Passeriformes | Dendrocolaptidae | *Lepidocolaptes* | *souleyetii* | 0.049 | 27.45 | 11.99 | 4.1 |
|  | Passeriformes | Dendrocolaptidae | *Lepidocolaptes* | *angustirostris* |  |  |  | 4.1 |
| 410 | Passeriformes | Dendrocolaptidae | *Lepidocolaptes* | *affinis* | 0.063 | 33.53 | 12.57 | 4.1 |
|  | Passeriformes | Dendrocolaptidae | *Lepidocolaptes* | *lacrymiger* |  |  |  | 4.1 |
| 411 | Passeriformes | Paridae | *Lophophanes* | *cristatus* | 0.063 | 12.13 | 16.29 | 4 |
|  | Passeriformes | Paridae | *Lophophanes* | *dichrous* |  |  |  | 4.2 |
| 412 | Passeriformes | Paridae | *Baeolophus* | *inornatus* | 0.047 | 15.94 | 1.08 | 4.4 |
|  | Passeriformes | Paridae | *Baeolophus* | *ridgwayi* |  |  |  | 4 |
| 413 | Passeriformes | Paridae | *Periparus* | *elegans* | 0.034 | 12.85 | 0 | 4.2 |
|  | Passeriformes | Paridae | *Periparus* | *amabilis* |  |  |  | 4.2 |
| 414 | Passeriformes | Paridae | *Cyanistes* | *caeruleus* | 0.052 | 13.28 | 27.99 | 4.4 |
|  | Passeriformes | Paridae | *Cyanistes* | *cyanus* |  |  |  | 4.2 |
| 415 | Passeriformes | Furnariidae | *Premnoplex* | *brunnescens* | 0.096 | 18 |  | 3.8 |
|  | Passeriformes | Furnariidae | *Premnoplex* | *tatei* |  |  |  | 3.8 |
| 416 | Passeriformes | Furnariidae | *Upucerthia* | *harterti* | 0.013 | 22.1 | 0 | 3.8 |
|  | Passeriformes | Furnariidae | *Upucerthia* | *certhioides* |  |  |  | 3.8 |
| 417 | Passeriformes | Furnariidae | *Upucerthia* | *dumetaria* | 0.113 | 48.97 | 0.82 | 3.8 |
|  | Passeriformes | Furnariidae | *Upucerthia* | *serrana* |  |  |  | 3.8 |
| 418 | Passeriformes | Furnariidae | *Upucerthia* | *ruficaudus* | 0.095 | 36.3 |  | 3.8 |
|  | Passeriformes | Furnariidae | *Upucerthia* | *andaecola* |  |  |  | 3.8 |
| 419 | Passeriformes | Furnariidae | *Geositta* | *rufipennis* | 0.037 | 30.04 | 24.74 | 3.8 |
|  | Passeriformes | Furnariidae | *Geositta* | *punensis* |  |  |  | 3.8 |
| 420 | Passeriformes | Furnariidae | *Geositta* | *maritima* | 0.087 | 29.75 | 61.63 | 3.8 |
|  | Passeriformes | Furnariidae | *Geositta* | *isabellina* |  |  |  | 3.8 |
| 421 | Passeriformes | Corvidae | *Calocitta* | *colliei* | 0.069 | 222 | 10.26 | 6.7 |
|  | Passeriformes | Corvidae | *Calocitta* | *formosa* |  |  |  | 6.7 |
| 422 | Passeriformes | Corvidae | *Pyrrhocorax* | *pyrrhocorax* | 0.12 | 244.76 | 23.92 | 11 |
|  | Passeriformes | Corvidae | *Pyrrhocorax* | *graculus* |  |  |  | 10.3 |
| 423 | Passeriformes | Corvidae | *Perisoreus* | *infaustus* | 0.055 | 93.2 | 17.25 | 7.1 |
|  | Passeriformes | Corvidae | *Perisoreus* | *internigrans* |  |  |  | 7.1 |
| 424 | Passeriformes | Corvidae | *Cyanopica* | *cyanus* | 0.07 | 96 |  | 6 |
|  | Passeriformes | Corvidae | *Cyanopica* | *cooki* |  |  |  | 6 |
| 425 | Passeriformes | Corvidae | *Pica* | *pica* | 0.035 | 192.14 | 14.16 | 7.1 |
|  | Passeriformes | Corvidae | *Pica* | *hudsonia* |  |  |  | 7.1 |
| 426 | Passeriformes | Corvidae | *Cyanocitta* | *cristata* | 0.119 | 108 | 31.25 | 6.8 |
|  | Passeriformes | Corvidae | *Cyanocitta* | *stelleri* |  |  |  | 7 |
| 427 | Passeriformes | Corvidae | *Cyanocorax* | *chrysops* | 0.045 | 156 | 12.05 | 6.1 |
|  | Passeriformes | Corvidae | *Cyanocorax* | *cyanopogon* |  |  |  | 6.1 |
| 428 | Passeriformes | Corvidae | *Cyanocorax* | *cyanomelas* | 0.019 | 192.5 | 14.01 | 6.1 |
|  | Passeriformes | Corvidae | *Cyanocorax* | *cristatellus* |  |  |  | 6.1 |
| 429 | Passeriformes | Corvidae | *Cyanocorax* | *melanocyaneus* | 0.06 | 109.5 | 14.41 | 6.1 |
|  | Passeriformes | Corvidae | *Cyanocorax* | *yucatanicus* |  |  |  | 6.1 |
| 430 | Passeriformes | Mimidae | *Toxostoma* | *crissale* | 0.065 | 62.3 | 1.28 | 4.2 |
|  | Passeriformes | Mimidae | *Toxostoma* | *lecontei* |  |  |  | 4.2 |
| 431 | Passeriformes | Mimidae | *Toxostoma* | *longirostre* | 0.054 | 60.2 | 21.89 | 4.2 |
|  | Passeriformes | Mimidae | *Toxostoma* | *guttatum* |  |  |  | 4.2 |
| 432 | Passeriformes | Laniidae | *Lanius* | *excubitoroides* | 0.084 | 63.83 | 27.27 | 3.9 |
|  | Passeriformes | Laniidae | *Lanius* | *cabanisi* |  |  |  | 3.9 |
| 433 | Passeriformes | Laniidae | *Lanius* | *collurio* | 0.032 | 27.37 | 11.42 | 4 |
|  | Passeriformes | Laniidae | *Lanius* | *isabellinus* |  |  |  | 3.9 |
| 434 | Passeriformes | Laniidae | *Lanius* | *bucephalus* | 0.028 | 37.2 | 19.22 | 3.9 |
|  | Passeriformes | Laniidae | *Lanius* | *cristatus* |  |  |  | 3.9 |
| 435 | Passeriformes | Dendrocolaptidae | *Xiphorhynchus* | *picus* | 0.084 | 41.3 | 11.42 | 4.8 |
|  | Passeriformes | Dendrocolaptidae | *Xiphorhynchus* | *kienerii* |  |  |  | 4.8 |
| 436 | Passeriformes | Dendrocolaptidae | *Xiphorhynchus* | *flavigaster* | 0.036 | 48.95 | 17.61 | 4.8 |
|  | Passeriformes | Dendrocolaptidae | *Xiphorhynchus* | *lachrymosus* |  |  |  | 4.8 |
| 437 | Passeriformes | Dendrocolaptidae | *Xiphorhynchus* | *occelatus* | 0.039 | 35.43 | 6.35 | 4.8 |
|  | Passeriformes | Dendrocolaptidae | *Xiphorhynchus* | *pardalotus* |  |  |  | 5.5 |
| 438 | Passeriformes | Muscicapidae | *Myrmecocichla* | *nigra* | 0.093 | 40.3 |  | 3.8 |
|  | Passeriformes | Muscicapidae | *Myrmecocichla* | *melaena* |  |  |  | 3.8 |
| 439 | Passeriformes | Muscicapidae | *Rhyacornis* | *fuliginosa* | 0.036 | 21.27 | 11.3 | 3.8 |
|  | Passeriformes | Muscicapidae | *Rhyacornis* | *bicolor* |  |  |  | 3.8 |
| 440 | Passeriformes | Muscicapidae | *Niltava* | *vivida* | 0.094 | 33 |  | 3.2 |
|  | Passeriformes | Muscicapidae | *Niltava* | *davidi* |  |  |  | 3.2 |
| 441 | Passeriformes | Muscicapidae | *Cercotrichas* | *hartlaubi* | 0.09 | 17.4 | 14.06 | 3.8 |
|  | Passeriformes | Muscicapidae | *Cercotrichas* | *leucophrys* |  |  |  | 3.8 |
| 442 | Passeriformes | Muscicapidae | *Cercotrichas* | *paena* | 0.139 | 20.05 | 3.43 | 3.8 |
|  | Passeriformes | Muscicapidae | *Cercotrichas* | *coryphaeus* |  |  |  | 3.8 |
| 443 | Passeriformes | Muscicapidae | *Cercotrichas* | *leucosticta* | 0.087 | 25.45 |  | 3.8 |
|  | Passeriformes | Muscicapidae | *Cercotrichas* | *barbata* |  |  |  | 3.8 |
| 444 | Passeriformes | Muscicapidae | *Muscicapa* | *striata* | 0.078 | 14.45 | 18.24 | 2.9 |
|  | Passeriformes | Muscicapidae | *Muscicapa* | *gambagae* |  |  |  | 2.9 |
| 445 | Passeriformes | Muscicapidae | *Muscicapa* | *aquatica* | 0.065 | 14.4 | 33.53 | 2.9 |
|  | Passeriformes | Muscicapidae | *Muscicapa* | *cassini* |  |  |  | 2.9 |
| 446 | Passeriformes | Muscicapidae | *Muscicapa* | *caerulescens* | 0.1 | 16.23 | 11.65 | 2.9 |
|  | Passeriformes | Muscicapidae | *Muscicapa* | *olivascens* |  |  |  | 2.9 |
| 447 | Passeriformes | Muscicapidae | *Cercomela* | *tractrac* | 0.043 | 19.45 | 12.98 | 3.8 |
|  | Passeriformes | Muscicapidae | *Cercomela* | *sinuata* |  |  |  | 3.8 |
| 448 | Passeriformes | Muscicapidae | *Oenanthe* | *finschii* | 0.02 | 24.98 | 18.03 | 4.1 |
|  | Passeriformes | Muscicapidae | *Oenanthe* | *lugens* |  |  |  | 4.1 |
| 449 | Passeriformes | Muscicapidae | *Oenanthe* | *bottae* | 0.013 | 24.65 | 32.31 | 4.1 |
|  | Passeriformes | Muscicapidae | *Oenanthe* | *isabellina* |  |  |  | 4.1 |
| 450 | Passeriformes | Muscicapidae | *Oenanthe* | *hispanica* | 0.003 | 17.25 | 22.16 | 4.1 |
|  | Passeriformes | Muscicapidae | *Oenanthe* | *pleschanka* |  |  |  | 4.1 |
| 451 | Passeriformes | Thamnophilidae | *Hylophylax* | *naevioides* | 0.078 | 16 | 20.22 | 5.9 |
|  | Passeriformes | Thamnophilidae | *Hylophylax* | *naevius* |  |  |  | 5.9 |
| 452 | Passeriformes | Thamnophilidae | *Drymophila* | *devillei* | 0.103 | 11.05 | 14.29 | 4.8 |
|  | Passeriformes | Thamnophilidae | *Drymophila* | *caudata* |  |  |  | 4.8 |
| 453 | Passeriformes | Thamnophilidae | *Rhegmatorhina* | *gymnops* | 0.027 | 29.95 | 9.24 | 4.8 |
|  | Passeriformes | Thamnophilidae | *Rhegmatorhina* | *hoffmannsi* |  |  |  | 4.8 |
| 454 | Passeriformes | Thamnophilidae | *Myrmeciza* | *nigricauda* | 0.089 | 24 |  | 4.8 |
|  | Passeriformes | Thamnophilidae | *Myrmeciza* | *berlepschi* |  |  |  | 4.8 |
| 455 | Passeriformes | Thamnophilidae | *Myrmeciza* | *melanoceps* | 0.031 | 40.25 | 8.33 | 4.8 |
|  | Passeriformes | Thamnophilidae | *Myrmeciza* | *goeldii* |  |  |  | 4.8 |
| 456 | Passeriformes | Thamnophilidae | *Myrmeciza* | *fortis* | 0.049 | 46.5 | 2.55 | 4.8 |
|  | Passeriformes | Thamnophilidae | *Myrmeciza* | *immaculata* |  |  |  | 4.8 |
| 457 | Passeriformes | Thamnophilidae | *Myrmeciza* | *castanea* | 0.037 | 16.25 | 3.03 | 4.8 |
|  | Passeriformes | Thamnophilidae | *Myrmeciza* | *hemimelaena* |  |  |  | 4.8 |
| 458 | Passeriformes | Thamnophilidae | *Myrmeciza* | *loricata* | 0.048 | 18.5 |  | 4.8 |
|  | Passeriformes | Thamnophilidae | *Myrmeciza* | *squamosa* |  |  |  | 4.8 |
| 459 | Passeriformes | Timaliidae | *Liocichla* | *phoenicea* | 0.015 | 49 |  | 5.5 |
|  | Passeriformes | Timaliidae | *Liocichla* | *omeiensis* |  |  |  | 5.5 |
| 460 | Passeriformes | Timaliidae | *Leiothrix* | *argentauris* | 0.082 | 23.73 | 24.65 | 5.6 |
|  | Passeriformes | Timaliidae | *Leiothrix* | *lutea* |  |  |  | 5.6 |
| 461 | Passeriformes | Uncertain | *Neomixis* | *tenella* | 0.081 | 7.75 | 13.25 | 4.4 |
|  | Passeriformes | Uncertain | *Neomixis* | *striatigula* |  |  |  | 4.4 |
| 462 | Passeriformes | Timaliidae | *Heterophasia* | *capistrata* | 0.097 | 36 | 17.26 | 4.9 |
|  | Passeriformes | Timaliidae | *Heterophasia* | *melanoleuca* |  |  |  | 4.9 |
| 463 | Passeriformes | Timaliidae | *Actinodura* | *egertoni* | 0.058 | 37.2 | 6.25 | 5.5 |
|  | Passeriformes | Timaliidae | *Actinodura* | *ramsayi* |  |  |  | 5.5 |
| 464 | Passeriformes | Timaliidae | *Actinodura* | *waldeni* | 0.009 | 18 |  | 5.5 |
|  | Passeriformes | Timaliidae | *Actinodura* | *souliei* |  |  |  | 5.5 |
| 465 | Passeriformes | Timaliidae | *Yuhina* | *castaniceps* | 0.032 | 11.95 | 2.48 | 5.7 |
|  | Passeriformes | Timaliidae | *Yuhina* | *everetti* |  |  |  | 5.7 |
| 466 | Passeriformes | Turdidae | *Entomodestes* | *coracinus* | 0.017 | 58.85 | 9.24 | 4.3 |
|  | Passeriformes | Turdidae | *Entomodestes* | *leucotis* |  |  |  | 4.3 |
| 467 | Passeriformes | Turdidae | *Stizorhina* | *fraseri* | 0.037 | 36.57 | 3.51 | 4.3 |
|  | Passeriformes | Turdidae | *Stizorhina* | *finschii* |  |  |  | 4.3 |
| 468 | Passeriformes | Turdidae | *Platycichla* | *flavipes* | 0.059 | 64.6 | 4.49 | 5.6 |
|  | Passeriformes | Turdidae | *Platycichla* | *leucops* |  |  |  | 6.1 |
| 469 | Passeriformes | Turdidae | *Sialia* | *sialis* | 0.05 | 26.8 | 3.82 | 3.9 |
|  | Passeriformes | Turdidae | *Sialia* | *mexicana* |  |  |  | 3.8 |
| 470 | Passeriformes | Muscicapidae | *Tarsiger* | *cyanurus* | 0.038 | 12.95 | 13.67 | 3.8 |
|  | Passeriformes | Muscicapidae | *Tarsiger* | *hyperythrus* |  |  |  | 3.8 |
| 471 | Passeriformes | Turdidae | *Myadestes* | *coloratus* | 0.042 | 29.12 | 0.09 | 4.5 |
|  | Passeriformes | Turdidae | *Myadestes* | *ralloides* |  |  |  | 4.5 |
| 472 | Passeriformes | Muscicapidae | *Sheppardia* | *aurantiithorax* | 0.036 | 18.4 |  | 3.8 |
|  | Passeriformes | Muscicapidae | *Sheppardia* | *lowei* |  |  |  | 3.8 |
| 473 | Passeriformes | Muscicapidae | *Sheppardia* | *gabela* | 0.07 | 13.9 | 34.52 | 3.8 |
|  | Passeriformes | Muscicapidae | *Sheppardia* | *aequatorialis* |  |  |  | 3.8 |
| 474 | Passeriformes | Muscicapidae | *Stiphrornis* | *xanthogaster* | 0.029 | 15.6 | 0 | 3.8 |
|  | Passeriformes | Muscicapidae | *Stiphrornis* | *sanghensis* |  |  |  | 3.8 |
| 475 | Passeriformes | Muscicapidae | *Stiphrornis* | *erythrothorax* | 0.045 | 15.6 |  | 3.8 |
|  | Passeriformes | Muscicapidae | *Stiphrornis* | *gabonensis* |  |  |  | 3.8 |
| 476 | Passeriformes | Turdidae | *Alethe* | *poliophrys* | 0.095 | 34.48 | 4.09 | 6.5 |
|  | Passeriformes | Turdidae | *Alethe* | *poliocephala* |  |  |  | 6.5 |
| 477 | Passeriformes | Turdidae | *Alethe* | *fuelleborni* | 0.082 | 46.65 | 20.58 | 6.5 |
|  | Passeriformes | Turdidae | *Alethe* | *choloensis* |  |  |  | 6.5 |
| 478 | Passeriformes | Thamnophilidae | *Thamnophilus* | *aethiops* | 0.053 | 25.7 |  | 4.9 |
|  | Passeriformes | Thamnophilidae | *Thamnophilus* | *aroyae* |  |  |  | 4.9 |
| 479 | Passeriformes | Thamnophilidae | *Thamnophilus* | *nigrocinereus* | 0.014 | 28.75 | 8.33 | 4.9 |
|  | Passeriformes | Thamnophilidae | *Thamnophilus* | *cryptoleucus* |  |  |  | 4.9 |
| 480 | Passeriformes | Thamnophilidae | *Thamnophilus* | *punctatus* | 0.05 | 20.45 | 9.77 | 4.9 |
|  | Passeriformes | Thamnophilidae | *Thamnophilus* | *stictocephalus* |  |  |  | 4.9 |
| 481 | Passeriformes | Thamnophilidae | *Thamnophilus* | *sticturus* | 0.017 | 19.55 | 12.92 | 4.9 |
|  | Passeriformes | Thamnophilidae | *Thamnophilus* | *pelzelni* |  |  |  | 4.9 |
| 482 | Passeriformes | Thamnophilidae | *Thamnophilus* | *nigriceps* | 0.029 | 22.9 |  | 4.9 |
|  | Passeriformes | Thamnophilidae | *Thamnophilus* | *praecox* |  |  |  | 4.9 |
| 483 | Passeriformes | Thamnophilidae | *Thamnophilus* | *torquatus* | 0.05 | 20.75 | 3.32 | 4.9 |
|  | Passeriformes | Thamnophilidae | *Thamnophilus* | *ruficapillus* |  |  |  | 4.9 |
| 484 | Passeriformes | Thamnophilidae | *Thamnophilus* | *tenuepunctatus* | 0.008 | 23.3 |  | 4.9 |
|  | Passeriformes | Thamnophilidae | *Thamnophilus* | *palliatus* |  |  |  | 4.9 |
| 485 | Passeriformes | Thamnophilidae | *Thamnophilus* | *bridgesi* | 0.063 | 25.3 | 12.59 | 4.9 |
|  | Passeriformes | Thamnophilidae | *Thamnophilus* | *atrinucha* |  |  |  | 4.9 |
| 486 | Passeriformes | Muscicapidae | *Pseudocossyphus* | *sharpei* | 0.01 | 25.53 | 5.52 | 3.8 |
|  | Passeriformes | Muscicapidae | *Pseudocossyphus* | *bensoni* |  |  |  | 3.8 |
| 487 | Passeriformes | Muscicapidae | *Monticola* | *pretoriae* | 0.013 | 33 |  | 3.8 |
|  | Passeriformes | Muscicapidae | *Monticola* | *brevipes* |  |  |  | 3.8 |
| 488 | Passeriformes | Muscicapidae | *Monticola* | *gularis* | 0.09 | 43.9 | 34.96 | 3.8 |
|  | Passeriformes | Muscicapidae | *Monticola* | *rufiventris* |  |  |  | 3.8 |
| 489 | Passeriformes | Muscicapidae | *Monticola* | *rupestris* | 0.013 | 52.9 | 27.69 | 3.8 |
|  | Passeriformes | Muscicapidae | *Monticola* | *angolensis* |  |  |  | 3.8 |
| 490 | Passeriformes | Muscicapidae | *Erithacus* | *akahige* | 0.073 | 20.45 | 28.87 | 6 |
|  | Passeriformes | Muscicapidae | *Erithacus* | *komadori* |  |  |  | 6 |
| 491 | Passeriformes | Turdidae | *Turdus* | *serranus* | 0.041 | 68.8 | 37.93 | 7.7 |
|  | Passeriformes | Turdidae | *Turdus* | *nigriceps* |  |  |  | 6.3 |
| 492 | Passeriformes | Turdidae | *Turdus* | *assimilis* | 0.067 | 62.1 | 23.08 | 6.7 |
|  | Passeriformes | Turdidae | *Turdus* | *albicollis* |  |  |  | 5.3 |
| 493 | Passeriformes | Turdidae | *Turdus* | *ignobilis* | 0.008 | 65.9 | 8.79 | 7.5 |
|  | Passeriformes | Turdidae | *Turdus* | *maranonicus* |  |  |  | 6.6 |
| 494 | Passeriformes | Turdidae | *Turdus* | *fumigatus* | 0.042 | 70.52 | 4.21 | 5.7 |
|  | Passeriformes | Turdidae | *Turdus* | *hauxwelli* |  |  |  | 7 |
| 495 | Passeriformes | Turdidae | *Turdus* | *haplochrous* | 0.033 | 73.95 | 23.93 | 6.1 |
|  | Passeriformes | Turdidae | *Turdus* | *nudigenis* |  |  |  | 5.8 |
| 496 | Passeriformes | Turdidae | *Turdus* | *cardis* | 0.012 | 61.53 | 11.62 | 6.1 |
|  | Passeriformes | Turdidae | *Turdus* | *hortulorum* |  |  |  | 6.1 |
| 497 | Passeriformes | Turdidae | *Turdus* | *unicolor* | 0.005 | 69.6 | 14.4 | 6.1 |
|  | Passeriformes | Turdidae | *Turdus* | *dissimilis* |  |  |  | 6.1 |
| 498 | Passeriformes | Turdidae | *Turdus* | *obscurus* | 0.018 | 67.35 | 13.18 | 6.1 |
|  | Passeriformes | Turdidae | *Turdus* | *pallidus* |  |  |  | 6.1 |
| 499 | Passeriformes | Turdidae | *Turdus* | *ruficollis* | 0.011 | 80.45 | 6.14 | 6.1 |
|  | Passeriformes | Turdidae | *Turdus* | *naumanni* |  |  |  | 6.1 |
| 500 | Passeriformes | Turdidae | *Turdus* | *plumbeus* | 0.047 | 78.65 | 7.94 | 7 |
|  | Passeriformes | Turdidae | *Turdus* | *aurantius* |  |  |  | 6.1 |
| 501 | Passeriformes | Turdidae | *Turdus* | *nigrescens* | 0.055 | 84.2 | 24.58 | 6.1 |
|  | Passeriformes | Turdidae | *Turdus* | *infuscatus* |  |  |  | 6.1 |
| 502 | Passeriformes | Turdidae | *Turdus* | *rufitorques* | 0.006 | 75.25 | 8.28 | 7.5 |
|  | Passeriformes | Turdidae | *Turdus* | *migratorius* |  |  |  | 5.3 |
| 503 | Passeriformes | Turdidae | *Turdus* | *jamaicensis* | 0.054 | 67.05 | 21.2 | 5.8 |
|  | Passeriformes | Turdidae | *Turdus* | *swalesi* |  |  |  | 6.1 |
| 504 | Passeriformes | Icteridae | *Icterus* | *galbula* | 0.002 | 32.9 | 0.45 | 5 |
|  | Passeriformes | Icteridae | *Icterus* | *abeillei* |  |  |  | 5.1 |
| 505 | Passeriformes | Icteridae | *Icterus* | *nigrogularis* | 0.029 | 50.27 | 27.31 | 5.1 |
|  | Passeriformes | Icteridae | *Icterus* | *gularis* |  |  |  | 5.1 |
| 506 | Passeriformes | Icteridae | *Icterus* | *bullockii* | 0.032 | 31.85 | 31.93 | 5 |
|  | Passeriformes | Icteridae | *Icterus* | *pustulatus* |  |  |  | 5.1 |
| 507 | Passeriformes | Icteridae | *Icterus* | *chrysater* | 0.016 | 47.8 | 20.97 | 5.1 |
|  | Passeriformes | Icteridae | *Icterus* | *graduacauda* |  |  |  | 5.1 |
| 508 | Passeriformes | Icteridae | *Icterus* | *icterus* | 0.028 | 62.67 | 39.97 | 5.1 |
|  | Passeriformes | Icteridae | *Icterus* | *jamaicaii* |  |  |  | 5.1 |
| 509 | Passeriformes | Thraupidae | *Tangara* | *cayana* | 0.007 | 22.95 | 35.48 | 4.9 |
|  | Passeriformes | Thraupidae | *Tangara* | *cucullata* |  |  |  | 4.9 |
| 510 | Passeriformes | Thraupidae | *Tangara* | *inornata* | 0.05 | 19.25 | 12.2 | 4.9 |
|  | Passeriformes | Thraupidae | *Tangara* | *mexicana* |  |  |  | 4.9 |
| 511 | Passeriformes | Thraupidae | *Tangara* | *velia* | 0.033 | 21.95 | 8.3 | 4.9 |
|  | Passeriformes | Thraupidae | *Tangara* | *callophrys* |  |  |  | 4.9 |
| 512 | Passeriformes | Thraupidae | *Tangara* | *johannae* | 0.068 | 19 |  | 4.9 |
|  | Passeriformes | Thraupidae | *Tangara* | *schrankii* |  |  |  | 4.9 |
| 513 | Passeriformes | Thraupidae | *Tangara* | *florida* | 0.036 | 20.65 | 12.27 | 4.9 |
|  | Passeriformes | Thraupidae | *Tangara* | *icterocephala* |  |  |  | 4.9 |
| 514 | Passeriformes | Thraupidae | *Tangara* | *fastuosa* | 0.024 | 18.7 |  | 4.9 |
|  | Passeriformes | Thraupidae | *Tangara* | *seledon* |  |  |  | 4.9 |
| 515 | Passeriformes | Thraupidae | *Tangara* | *cyanocephala* | 0.04 | 19.2 | 11.76 | 4.9 |
|  | Passeriformes | Thraupidae | *Tangara* | *desmaresti* |  |  |  | 4.9 |
| 516 | Passeriformes | Thraupidae | *Tangara* | *dowii* | 0.057 | 21 |  | 4.9 |
|  | Passeriformes | Thraupidae | *Tangara* | *fucosa* |  |  |  | 4.9 |
| 517 | Passeriformes | Thraupidae | *Tangara* | *argyrofenges* | 0.002 | 19.6 | 5.94 | 4.9 |
|  | Passeriformes | Thraupidae | *Tangara* | *heinei* |  |  |  | 4.9 |
| 518 | Passeriformes | Thraupidae | *Tangara* | *cyanicollis* | 0.044 | 18.5 | 15 | 4.9 |
|  | Passeriformes | Thraupidae | *Tangara* | *larvata* |  |  |  | 4.9 |
| 519 | Passeriformes | Emberizidae | *Plectrophenax* | *nivalis* | 0.001 | 48.35 | 22.57 | 3.9 |
|  | Passeriformes | Emberizidae | *Plectrophenax* | *hyperboreus* |  |  |  | 3.9 |
| 520 | Passeriformes | Cardinalidae | *Caryothraustes* | *poliogaster* | 0.056 | 38.15 | 17.46 | 4.1 |
|  | Passeriformes | Cardinalidae | *Caryothraustes* | *canadensis* |  |  |  | 4.1 |
| 521 | Passeriformes | Thraupidae | *Mitrospingus* | *cassinii* | 0.081 | 40.85 | 2.18 | 3.7 |
|  | Passeriformes | Thraupidae | *Mitrospingus* | *oleagineus* |  |  |  | 3.7 |
| 522 | Passeriformes | Icteridae | *Euphagus* | *carolinus* | 0.04 | 61.2 | 4.63 | 4.2 |
|  | Passeriformes | Icteridae | *Euphagus* | *cyanocephalus* |  |  |  | 4.2 |
| 523 | Passeriformes | Thraupidae | *Lophospingus* | *pusillus* | 0.03 | 15.6 | 19.27 | 3.8 |
|  | Passeriformes | Thraupidae | *Lophospingus* | *griseocristatus* |  |  |  | 3.8 |
| 524 | Passeriformes | Icteridae | *Pseudoleistes* | *guirahuro* | 0.034 | 83.25 | 7.63 | 4.6 |
|  | Passeriformes | Icteridae | *Pseudoleistes* | *virescens* |  |  |  | 4.6 |
| 525 | Passeriformes | Thraupidae | *Calyptophilus* | *tertius* | 0.086 | 40.65 | 34.76 | 3.7 |
|  | Passeriformes | Thraupidae | *Calyptophilus* | *frugivorus* |  |  |  | 3.7 |
| 526 | Passeriformes | Fringillidae | *Eophona* | *migratoria* | 0.054 | 57.93 | 41.38 | 4.3 |
|  | Passeriformes | Fringillidae | *Eophona* | *personata* |  |  |  | 4.3 |
| 527 | Passeriformes | Emberizidae | *Pselliophorus* | *tibialis* | 0.007 | 30 |  | 3.8 |
|  | Passeriformes | Emberizidae | *Pselliophorus* | *luteoviridis* |  |  |  | 3.8 |
| 528 | Passeriformes | Parulidae | *Ergaticus* | *ruber* | 0.019 | 9.05 | 19 | 3.9 |
|  | Passeriformes | Parulidae | *Ergaticus* | *versicolor* |  |  |  | 3.9 |
| 529 | Passeriformes | Thraupidae | *Heterospingus* | *rubifrons* | 0.033 | 38.4 | 2.06 | 3.7 |
|  | Passeriformes | Thraupidae | *Heterospingus* | *xanthopygius* |  |  |  | 3.7 |
| 530 | Passeriformes | Cardinalidae | *Cyanocompsa* | *cyanoides* | 0.06 | 30 | 15.38 | 4.2 |
|  | Passeriformes | Cardinalidae | *Cyanocompsa* | *brissonii* |  |  |  | 4.2 |
| 531 | Passeriformes | Icteridae | *Dives* | *dives* | 0.047 | 85.57 | 20.07 | 4.6 |
|  | Passeriformes | Icteridae | *Dives* | *warszewiczi* |  |  |  | 4.6 |
| 532 | Passeriformes | Thraupidae | *Diglossopis* | *caerulescens* | 0.065 | 15.75 | 15.25 | 3.7 |
|  | Passeriformes | Thraupidae | *Diglossopis* | *cyanea* |  |  |  | 3.7 |
| 533 | Passeriformes | Thraupidae | *Chlorochrysa* | *calliparaea* | 0.037 | 17.8 | 8.6 | 3.7 |
|  | Passeriformes | Thraupidae | *Chlorochrysa* | *nitidissima* |  |  |  | 3.7 |
| 534 | Passeriformes | Thraupidae | *Creurgops* | *verticalis* | 0.087 | 21.5 | 20.83 | 3.7 |
|  | Passeriformes | Thraupidae | *Creurgops* | *dentatus* |  |  |  | 3.7 |
| 535 | Passeriformes | Emberizidae | *Arremonops* | *rufivirgatus* | 0.047 | 24.1 | 18.07 | 3.3 |
|  | Passeriformes | Emberizidae | *Arremonops* | *chloronotus* |  |  |  | 3.3 |
| 536 | Passeriformes | Emberizidae | *Melozone* | *kieneri* | 0.061 | 34.65 | 20.93 | 3.8 |
|  | Passeriformes | Emberizidae | *Melozone* | *biarcuata* |  |  |  | 3.8 |
| 537 | Passeriformes | Thraupidae | *Conirostrum* | *bicolor* | 0.056 | 10.5 |  | 3.7 |
|  | Passeriformes | Thraupidae | *Conirostrum* | *margaritae* |  |  |  | 3.7 |
| 538 | Passeriformes | Thraupidae | *Conirostrum* | *sitticolor* | 0.06 | 13.4 | 20.13 | 3.7 |
|  | Passeriformes | Thraupidae | *Conirostrum* | *albifrons* |  |  |  | 3.7 |
| 539 | Passeriformes | Thraupidae | *Haplospiza* | *rustica* | 0.088 | 15.4 | 1.92 | 3.8 |
|  | Passeriformes | Thraupidae | *Haplospiza* | *unicolor* |  |  |  | 3.8 |
| 540 | Passeriformes | Thraupidae | *Bangsia* | *edwardsi* | 0.017 | 39.35 | 11.27 | 3.7 |
|  | Passeriformes | Thraupidae | *Bangsia* | *aureocincta* |  |  |  | 3.7 |
| 541 | Passeriformes | Thraupidae | *Coryphospingus* | *pileatus* | 0.068 | 14.93 | 4.9 | 3.8 |
|  | Passeriformes | Thraupidae | *Coryphospingus* | *cucullatus* |  |  |  | 3.8 |
| 542 | Passeriformes | Thraupidae | *Lanio* | *aurantius* | 0.012 | 37.5 | 12.5 | 3.7 |
|  | Passeriformes | Thraupidae | *Lanio* | *leucothorax* |  |  |  | 3.7 |
| 543 | Passeriformes | Thraupidae | *Loxigilla* | *portoricensis* | 0.076 | 26.28 | 30.43 | 4.1 |
|  | Passeriformes | Thraupidae | *Loxigilla* | *violacea* |  |  |  | 4.1 |
| 544 | Passeriformes | Cardinalidae | *Cardinalis* | *cardinalis* | 0.045 | 42.65 |  | 4.9 |
|  | Passeriformes | Cardinalidae | *Cardinalis* | *phoeniceus* |  |  |  | 4.9 |
| 545 | Passeriformes | Thraupidae | *Anisognathus* | *lacrymosus* | 0.048 | 32.5 | 8.82 | 3.7 |
|  | Passeriformes | Thraupidae | *Anisognathus* | *igniventris* |  |  |  | 3.7 |
| 546 | Passeriformes | Thraupidae | *Buthraupis* | *montana* | 0.06 | 79.35 | 34.69 | 3.7 |
|  | Passeriformes | Thraupidae | *Buthraupis* | *eximia* |  |  |  | 3.7 |
| 547 | Passeriformes | Emberizidae | *Ammodramus* | *nelsoni* | 0.008 | 16.8 | 21.21 | 3.9 |
|  | Passeriformes | Emberizidae | *Ammodramus* | *caudacutus* |  |  |  | 3.8 |
| 548 | Passeriformes | Icteridae | *Molothrus* | *bonariensis* | 0.015 | 39.68 | 9.78 | 4.6 |
|  | Passeriformes | Icteridae | *Molothrus* | *ater* |  |  |  | 4.6 |
| 549 | Passeriformes | Thraupidae | *Cyanerpes* | *caerulesus* | 0.063 | 13 | 14.29 | 3.3 |
|  | Passeriformes | Thraupidae | *Cyanerpes* | *cyaneus* |  |  |  | 3.3 |
| 550 | Passeriformes | Emberizidae | *Aimophila* | *cassinii* | 0.055 | 19.03 | 1.36 | 3.7 |
|  | Passeriformes | Emberizidae | *Aimophila* | *aestivalis* |  |  |  | 3.7 |
| 551 | Passeriformes | Emberizidae | *Aimophila* | *humeralis* | 0.056 | 23.05 | 7.11 | 3.7 |
|  | Passeriformes | Emberizidae | *Aimophila* | *mystacalis* |  |  |  | 3.7 |
| 552 | Passeriformes | Emberizidae | *Aimophila* | *stolzmanni* | 0.079 | 24.5 | 13.45 | 3.7 |
|  | Passeriformes | Emberizidae | *Aimophila* | *strigiceps* |  |  |  | 3.7 |
| 553 | Passeriformes | Cardinalidae | *Passerina* | *caerulea* | 0.052 | 21.45 | 43.43 | 4.2 |
|  | Passeriformes | Cardinalidae | *Passerina* | *amoena* |  |  |  | 4.1 |
| 554 | Passeriformes | Cardinalidae | *Passerina* | *versicolor* | 0.031 | 14.23 | 17.04 | 4.2 |
|  | Passeriformes | Cardinalidae | *Passerina* | *ciris* |  |  |  | 4.4 |
| 555 | Passeriformes | Parulidae | *Parula* | *gutteralis* | 0.008 | 9.25 | 5.26 | 3 |
|  | Passeriformes | Parulidae | *Parula* | *superciliosa* |  |  |  | 3 |
| 556 | Passeriformes | Parulidae | *Parula* | *americana* | 0.019 | 7.48 | 12.29 | 3 |
|  | Passeriformes | Parulidae | *Parula* | *pitiayumi* |  |  |  | 3 |
| 557 | Passeriformes | Thraupidae | *Ramphocelus* | *flammigerus* | 0.024 | 33 |  | 3.7 |
|  | Passeriformes | Thraupidae | *Ramphocelus* | *icteronotus* |  |  |  | 3.7 |
| 558 | Passeriformes | Thraupidae | *Ramphocelus* | *melanogaster* | 0.005 | 26.35 | 9.75 | 3.7 |
|  | Passeriformes | Thraupidae | *Ramphocelus* | *carbo* |  |  |  | 3.7 |
| 559 | Passeriformes | Thraupidae | *Diglossa* | *baritula* | 0.011 | 9.6 | 8 | 3.7 |
|  | Passeriformes | Thraupidae | *Diglossa* | *plumbea* |  |  |  | 3.7 |
| 560 | Passeriformes | Thraupidae | *Diglossa* | *gloriosissima* | 0.028 | 15 | 12.5 | 3.7 |
|  | Passeriformes | Thraupidae | *Diglossa* | *lafresnayii* |  |  |  | 3.7 |
| 561 | Passeriformes | Thraupidae | *Thraupis* | *cyanocephala* | 0.1 | 36 | 0 | 4 |
|  | Passeriformes | Thraupidae | *Thraupis* | *bonariensis* |  |  |  | 4 |
| 562 | Passeriformes | Thraupidae | *Thraupis* | *ornata* | 0.018 | 36 | 15.38 | 4 |
|  | Passeriformes | Thraupidae | *Thraupis* | *palmarum* |  |  |  | 4 |
| 563 | Passeriformes | Thraupidae | *Thraupis* | *episcopus* | 0.028 | 33.5 | 8.57 | 4 |
|  | Passeriformes | Thraupidae | *Thraupis* | *sayaca* |  |  |  | 4 |
| 564 | Passeriformes | Thraupidae | *Tiaris* | *obscurus* | 0.037 | 12.25 | 15.79 | 3.4 |
|  | Passeriformes | Thraupidae | *Tiaris* | *fuliginosus* |  |  |  | 3.4 |
| 565 | Passeriformes | Emberizidae | *Pipilo* | *crissalis* | 0.022 | 49.4 | 13.06 | 4.3 |
|  | Passeriformes | Emberizidae | *Pipilo* | *aberti* |  |  |  | 4.2 |
| 566 | Passeriformes | Emberizidae | *Pipilo* | *maculatus* | 0.01 | 39.6 | 1.87 | 4.1 |
|  | Passeriformes | Emberizidae | *Pipilo* | *erythrophthalmus* |  |  |  | 4.1 |
| 567 | Passeriformes | Icteridae | *Psarocolius* | *atrovirens* | 0.052 | 296 | 65.45 | 4.6 |
|  | Passeriformes | Icteridae | *Psarocolius* | *angustifrons* |  |  |  | 4.6 |
| 568 | Passeriformes | Thraupidae | *Poospiza* | *ornata* | 0.049 | 11.8 |  | 3.8 |
|  | Passeriformes | Thraupidae | *Poospiza* | *boliviana* |  |  |  | 3.8 |
| 569 | Passeriformes | Thraupidae | *Compsospiza* | *garleppi* | 0.068 | 32.5 |  | 3.8 |
|  | Passeriformes | Thraupidae | *Compsospiza* | *baeri* |  |  |  | 3.8 |
| 570 | Passeriformes | Thraupidae | *Tachyphonus* | *coronatus* | 0.058 | 31.85 | 14.83 | 3.7 |
|  | Passeriformes | Thraupidae | *Tachyphonus* | *rufus* |  |  |  | 3.5 |
| 571 | Passeriformes | Thraupidae | *Tachyphonus* | *cristatus* | 0.082 | 18.9 | 1.05 | 3.7 |
|  | Passeriformes | Thraupidae | *Tachyphonus* | *rufiventer* |  |  |  | 3.7 |
| 572 | Passeriformes | Icteridae | *Cacicus* | *chrysonotus* | 0.025 | 36.45 |  | 4.6 |
|  | Passeriformes | Icteridae | *Cacicus* | *leucoramphus* |  |  |  | 4.6 |
| 573 | Passeriformes | Icteridae | *Cacicus* | *cela* | 0.063 | 86.4 | 2.4 | 4.6 |
|  | Passeriformes | Icteridae | *Cacicus* | *haemorrhous* |  |  |  | 4.6 |
| 574 | Passeriformes | Emberizidae | *Spizella* | *breweri* | 0 | 10.9 |  | 3.8 |
|  | Passeriformes | Emberizidae | *Spizella* | *taverneri* |  |  |  | 3.8 |
| 575 | Passeriformes | Emberizidae | *Spizella* | *pusilla* | 0.044 | 12.43 | 0.8 | 3.7 |
|  | Passeriformes | Emberizidae | *Spizella* | *wortheni* |  |  |  | 3.8 |
| 576 | Passeriformes | Thraupidae | *Chlorospingus* | *parvirostris* | 0.069 | 24.8 | 6.25 | 3.7 |
|  | Passeriformes | Thraupidae | *Chlorospingus* | *flavigularis* |  |  |  | 3.7 |
| 577 | Passeriformes | Thraupidae | *Chlorospingus* | *inornatus* | 0.049 | 23.5 | 32.14 | 3.7 |
|  | Passeriformes | Thraupidae | *Chlorospingus* | *semifuscus* |  |  |  | 3.7 |
| 578 | Passeriformes | Emberizidae | *Paroaria* | *coronata* | 0.081 | 36.3 | 12.29 | 3.8 |
|  | Passeriformes | Emberizidae | *Paroaria* | *dominicana* |  |  |  | 3.8 |
| 579 | Passeriformes | Parulidae | *Geothlypis* | *aequinoctialis* | 0.071 | 13.85 | 10.27 | 4.6 |
|  | Passeriformes | Parulidae | *Geothlypis* | *poliocephala* |  |  |  | 4.6 |
| 580 | Passeriformes | Parulidae | *Geothlypis* | *nelsoni* | 0.005 | 10.95 | 0.91 | 4.6 |
|  | Passeriformes | Parulidae | *Geothlypis* | *flavovelata* |  |  |  | 4.6 |
| 581 | Passeriformes | Emberizidae | *Amphispiza* | *bilineata* | 0.109 | 16.18 | 20.9 | 3.5 |
|  | Passeriformes | Emberizidae | *Amphispiza* | *belli* |  |  |  | 3.5 |
| 582 | Passeriformes | Emberizidae | *Calcarius* | *mccownii* | 0.09 | 26.78 | 7.72 | 4 |
|  | Passeriformes | Emberizidae | *Calcarius* | *lapponicus* |  |  |  | 3.3 |
| 583 | Passeriformes | Emberizidae | *Calcarius* | *pictus* | 0.035 | 24.57 | 23.97 | 5.1 |
|  | Passeriformes | Emberizidae | *Calcarius* | *ornatus* |  |  |  | 4 |
| 584 | Passeriformes | Parulidae | *Dendroica* | *plumbea* | 0.082 | 10 | 1.98 | 3.6 |
|  | Passeriformes | Parulidae | *Dendroica* | *pharetra* |  |  |  | 3.6 |
| 585 | Passeriformes | Parulidae | *Dendroica* | *pityophila* | 0.05 | 9.89 | 32.09 | 3.6 |
|  | Passeriformes | Parulidae | *Dendroica* | *pinus* |  |  |  | 3.6 |
| 586 | Passeriformes | Parulidae | *Dendroica* | *pensylvanica* | 0.041 | 9.44 | 2.41 | 3.5 |
|  | Passeriformes | Parulidae | *Dendroica* | *petechia* |  |  |  | 3.6 |
| 587 | Passeriformes | Parulidae | *Dendroica* | *townsendi* | 0.008 | 9.37 | 14.9 | 3.4 |
|  | Passeriformes | Parulidae | *Dendroica* | *occidentalis* |  |  |  | 3.6 |
| 588 | Passeriformes | Parulidae | *Dendroica* | *graciae* | 0.009 | 8.4 | 6.9 | 3.6 |
|  | Passeriformes | Parulidae | *Dendroica* | *nigrescens* |  |  |  | 3.9 |
| 589 | Passeriformes | Fringillidae | *Fringilla* | *coelebs* | 0.05 | 24.74 | 27.51 | 5.7 |
|  | Passeriformes | Fringillidae | *Fringilla* | *teydea* |  |  |  | 5.7 |
| 590 | Passeriformes | Parulidae | *Myioborus* | *ornatus* | 0.009 | 11.5 | 3.42 | 3.5 |
|  | Passeriformes | Parulidae | *Myioborus* | *melanocephalus* |  |  |  | 3.5 |
| 591 | Passeriformes | Parulidae | *Myioborus* | *castaneocapillus* | 0.018 | 10.93 |  | 3.5 |
|  | Passeriformes | Parulidae | *Myioborus* | *cardonai* |  |  |  | 3.5 |
| 592 | Passeriformes | Fringillidae | *Carduelis* | *flavirostris* | 0.037 | 18.17 | 21.23 | 4.2 |
|  | Passeriformes | Fringillidae | *Carduelis* | *cannabina* |  |  |  | 4.1 |
| 593 | Passeriformes | Fringillidae | *Carduelis* | *flammea* | 0.003 | 13.39 | 5.59 | 4.5 |
|  | Passeriformes | Fringillidae | *Carduelis* | *hornemanni* |  |  |  | 4.2 |
| 594 | Passeriformes | Fringillidae | *Carduelis* | *spinoides* | 0.015 | 18.6 |  | 4.2 |
|  | Passeriformes | Fringillidae | *Carduelis* | *ambigua* |  |  |  | 4.2 |
| 595 | Passeriformes | Fringillidae | *Carduelis* | *magellanica* | 0.012 | 13.6 |  | 4.2 |
|  | Passeriformes | Fringillidae | *Carduelis* | *olivacea* |  |  |  | 4.2 |
| 596 | Passeriformes | Fringillidae | *Carduelis* | *tristis* | 0.042 | 11.64 | 27.19 | 4.1 |
|  | Passeriformes | Fringillidae | *Carduelis* | *psaltria* |  |  |  | 4.5 |
| 597 | Passeriformes | Thraupidae | *Piranga* | *bidentata* | 0.046 | 32.34 | 23.16 | 4.1 |
|  | Passeriformes | Thraupidae | *Piranga* | *ludoviciana* |  |  |  | 4.1 |
| 598 | Passeriformes | Thraupidae | *Piranga* | *leucoptera* | 0.082 | 25.95 | 55.43 | 4.1 |
|  | Passeriformes | Thraupidae | *Piranga* | *rubriceps* |  |  |  | 4.1 |
| 599 | Passeriformes | Emberizidae | *Melospiza* | *lincolnii* | 0.038 | 16.35 | 3.01 | 3.6 |
|  | Passeriformes | Emberizidae | *Melospiza* | *georgiana* |  |  |  | 3.8 |
| 600 | Cuculiformes | Cuculidae | *Crotophaga* | *ani* | 0.047 | 101.45 | 31.9 | 4.2 |
|  | Cuculiformes | Cuculidae | *Crotophaga* | *sulcirostris* |  |  |  | 4.2 |
| 601 | Cuculiformes | Cuculidae | *Geococcyx* | *californianus* | 0.096 | 245.33 | 52.13 | 3.5 |
|  | Cuculiformes | Cuculidae | *Geococcyx* | *velox* |  |  |  | 3.5 |
| 602 | Cuculiformes | Cuculidae | *Piaya* | *cayana* | 0.075 | 102 |  | 4.2 |
|  | Cuculiformes | Cuculidae | *Piaya* | *melanogaster* |  |  |  | 4.2 |
| 603 | Cuculiformes | Cuculidae | *Coua* | *cursor* | 0.08 | 131.5 | 28.1 | 4.2 |
|  | Cuculiformes | Cuculidae | *Coua* | *reynaudii* |  |  |  | 4.2 |
| 604 | Coraciiformes | Bucorvidae | *Bucorvus* | *abyssinicus* | 0.035 | 3845 | 5.81 | 29.8 |
|  | Coraciiformes | Bucorvidae | *Bucorvus* | *leadbeateri* |  |  |  | 29.8 |
| 605 | Coraciiformes | Bucerotidae | *Anorrhinus* | *tickelli* | 0.053 | 796 |  | 11.1 |
|  | Coraciiformes | Bucerotidae | *Anorrhinus* | *austeni* |  |  |  | 11.1 |
| 606 | Coraciiformes | Bucerotidae | *Buceros* | *bicornis* | 0.043 | 2589.25 | 14.95 | 18.4 |
|  | Coraciiformes | Bucerotidae | *Buceros* | *rhinoceros* |  |  |  | 18.4 |
| 607 | Coraciiformes | Bucerotidae | *Tockus* | *monteiri* | 0.054 | 227.5 | 50.82 | 9.7 |
|  | Coraciiformes | Bucerotidae | *Tockus* | *erythrorhynchus* |  |  |  | 9.7 |
| 608 | Coraciiformes | Brachypteraciidae | *Atelornis* | *pittoides* | 0.072 | 85.25 | 13.66 | 5.6 |
|  | Coraciiformes | Brachypteraciidae | *Atelornis* | *crossleyi* |  |  |  | 5.6 |
| 609 | Coraciiformes | Momotidae | *Momotus* | *mexicanus* | 0.066 | 105.68 | 34.55 | 3.6 |
|  | Coraciiformes | Momotidae | *Momotus* | *momota* |  |  |  | 3.6 |
| 610 | Coraciiformes | Todidae | *Todus* | *todus* | 0.145 | 6.15 | 7.81 | 4.1 |
|  | Coraciiformes | Todidae | *Todus* | *mexicanus* |  |  |  | 4.1 |
| 611 | Coraciiformes | Todidae | *Todus* | *angustirostris* | 0.105 | 6.7 | 21.33 | 4.1 |
|  | Coraciiformes | Todidae | *Todus* | *multicolor* |  |  |  | 4.1 |
| 612 | Musophagiformes | Musophagidae | *Musophaga* | *violacea* | 0.057 | 367.5 | 4 | 3.3 |
|  | Musophagiformes | Musophagidae | *Musophaga* | *rossae* |  |  |  | 3.3 |
| 613 | Musophagiformes | Musophagidae | *Corythaixoides* | *concolor* | 0.06 | 254 | 3.1 | 3 |
|  | Musophagiformes | Musophagidae | *Corythaixoides* | *personatus* |  |  |  | 3 |
| 614 | Musophagiformes | Musophagidae | *Crinifer* | *piscator* | 0.048 | 448.5 | 29.79 | 3 |
|  | Musophagiformes | Musophagidae | *Crinifer* | *zonurus* |  |  |  | 3 |
| 615 | Musophagiformes | Musophagidae | *Tauraco* | *schalowi* | 0.019 | 222.5 | 1.34 | 3.3 |
|  | Musophagiformes | Musophagidae | *Tauraco* | *hartlaubi* |  |  |  | 3.3 |
| 616 | Musophagiformes | Musophagidae | *Tauraco* | *fischeri* | 0.015 | 249.5 | 0.4 | 3.3 |
|  | Musophagiformes | Musophagidae | *Tauraco* | *livingstonii* |  |  |  | 3.3 |
| 617 | Musophagiformes | Musophagidae | *Tauraco* | *leucolophus* | 0.027 | 243.25 | 18.13 | 3.3 |
|  | Musophagiformes | Musophagidae | *Tauraco* | *erythrolophus* |  |  |  | 3.3 |
| 618 | Trogoniiformes | Trogonidae | *Trogon* | *curucui* | 0.062 | 52.75 | 4.63 | 7.3 |
|  | Trogoniiformes | Trogonidae | *Trogon* | *violaceus* |  |  |  | 7.3 |
| 619 | Trogoniiformes | Trogonidae | *Trogon* | *viridis* | 0.071 | 92.05 | 4.98 | 7.3 |
|  | Trogoniiformes | Trogonidae | *Trogon* | *bairdii* |  |  |  | 7.3 |
| 620 | Trogoniiformes | Trogonidae | *Trogon* | *melanocephalus* | 0.01 | 82.05 | 7.17 | 7.3 |
|  | Trogoniiformes | Trogonidae | *Trogon* | *citreolus* |  |  |  | 7.3 |
| 621 | Trogoniiformes | Trogonidae | *Trogon* | *melanurus* | 0.052 | 127.5 | 19.15 | 7.3 |
|  | Trogoniiformes | Trogonidae | *Trogon* | *massena* |  |  |  | 7.3 |
| 622 | Trogoniiformes | Trogonidae | *Trogon* | *elegans* | 0.125 | 62.35 | 24.12 | 7.3 |
|  | Trogoniiformes | Trogonidae | *Trogon* | *rufus* |  |  |  | 7.3 |
| 623 | Trogoniiformes | Trogonidae | *Trogon* | *collaris* | 0.114 | 63.8 | 1.25 | 7.3 |
|  | Trogoniiformes | Trogonidae | *Trogon* | *personatus* |  |  |  | 7.3 |
| 624 | Passeriformes | Hirundinidae | *Hirundo* | *aethiopica* | 0.041 | 15.55 | 25.28 | 4 |
|  | Passeriformes | Hirundinidae | *Hirundo* | *angolensis* |  |  |  | 4 |
| 625 | Casuariiformes | Casuariidae | *Casuarius* | *casuarius* | 0.038 | 39500 | 20.45 | 11.5 |
|  | Casuariiformes | Casuariidae | *Casuarius* | *bennetti* |  |  |  | 11.3 |
| 626 | Dinornithiformes | Apterygidae | *Apteryx* | *owenii* | 0.02 | 1649 | 39.51 | 9 |
|  | Dinornithiformes | Apterygidae | *Apteryx* | *haastii* |  |  |  | 8.8 |
| 627 | Dinornithiformes | Apterygidae | *Apteryx* | *mantelli* | 0.016 | 2330 | 0 | 8.8 |
|  | Dinornithiformes | Apterygidae | *Apteryx* | *rowi* |  |  |  | 8.8 |
| 628 | Sphenisciformes | Spheniscidae | *Aptenodytes* | *forsteri* | 0.07 | 22800.5 | 65.29 | 20.4 |
|  | Sphenisciformes | Spheniscidae | *Aptenodytes* | *patagonicus* |  |  |  | 12.7 |
| 629 | Sphenisciformes | Spheniscidae | *Spheniscus* | *humboldti* | 0.01 | 3155.75 | 55.87 | 10.6 |
|  | Sphenisciformes | Spheniscidae | *Spheniscus* | *mendiculus* |  |  |  | 11.2 |
| 630 | Sphenisciformes | Spheniscidae | *Spheniscus* | *demersus* | 0.016 | 3627.5 | 23.91 | 9.3 |
|  | Sphenisciformes | Spheniscidae | *Spheniscus* | *magellanicus* |  |  |  | 11.7 |
| 631 | Sphenisciformes | Spheniscidae | *Eudyptes* | *schlegeli* | 0.013 | 4370 | 5.35 | 14.1 |
|  | Sphenisciformes | Spheniscidae | *Eudyptes* | *chrysolophus* |  |  |  | 11.4 |
| 632 | Sphenisciformes | Spheniscidae | *Eudyptes* | *pachyrhynchus* | 0.018 | 3480 | 21.99 | 9.6 |
|  | Sphenisciformes | Spheniscidae | *Eudyptes* | *robustus* |  |  |  | 11.1 |
| 633 | Rheiformes | Rheidae | *Rhea* | *americana* | 0.065 | 23450 | 3.77 | 10.5 |
|  | Rheiformes | Rheidae | *Rhea* | *pennata* |  |  |  | 11 |
